# 3D Interaction Homology: Computational Titration of Aspartic Acid, Glutamic Acid and Histidine Can Create pH-Tunable Hydropathic Environment Maps

**DOI:** 10.3389/fmolb.2021.773385

**Published:** 2021-11-03

**Authors:** Noah B. Herrington, Glen E. Kellogg

**Affiliations:** ^1^ Department of Medicinal Chemistry and Institute for Structural Biology, Drug Discovery and Development, Virginia Commonwealth University, Richmond, VA, United States; ^2^ Center for the Study of Biological Complexity, Virginia Commonwealth University, Richmond, VA, United States

**Keywords:** ionizable residues, aspartic acid, glutamic acid, histidine, hydropathic interactions, solvent-accessible surface area, pKa

## Abstract

Aspartic acid, glutamic acid and histidine are ionizable residues occupying various protein environments and perform many different functions in structures. Their roles are tied to their acid/base equilibria, solvent exposure, and backbone conformations. We propose that the number of unique environments for ASP, GLU and HIS is quite limited. We generated maps of these residue's environments using a hydropathic scoring function to record the type and magnitude of interactions for each residue in a 2703-protein structural dataset. These maps are backbone-dependent and suggest the existence of new structural motifs for each residue type. Additionally, we developed an algorithm for tuning these maps to any pH, a potentially useful element for protein design and structure building. Here, we elucidate the complex interplay between secondary structure, relative solvent accessibility, and residue ionization states: the degree of protonation for ionizable residues increases with solvent accessibility, which in turn is notably dependent on backbone structure.

## Introduction

Proteins are largely composed of unique combinations of 20 possible amino acids, varying from tens to thousands of residues in length. Specific protein sequences organize themselves into unique and well-defined secondary structures that comprise much larger and more complex structures that ultimately determine their functions. This relationship between structure and function is important to grasp in order to understand how different features of biological targets can be exploited for treatments of various disease states.

### pH, pK_a_ and Protonation States

One important aspect of this relationship is the dependence of protein structure on pH and protonation states of constituent residues. Histidine (HIS), for example, has a nominal pK_a_ of 6.00 (Hunt, 2021), situated closely enough to physiological pH that its imidazole sidechain can act either as a cationic dual hydrogen bond donor or a neutral donor and acceptor depending on its local pH environment. That is, the resultant influence of a residue’s neighborhood, comprised of the hydrogen bond donors, acceptors, charged species, and etc. that influence the solution pH surrounding it (Di Russo et al., 2012). The importance of histidine’s protonation state in the so-called “catalytic triad” of serine, histidine, and aspartate in serine proteases was shown decades ago for trypsin (Kasserra and Laidler, 1969; Antonino and Ascenzi, 1981). The pH-dependence of protein function is a well-established principle and has promoted extensive research into identifying optimum pH for activity of various other macromolecules (Talley and Alexov, 2010).

The pK_a_s of aspartic acid (ASP) and glutamic acid (GLU) when isolated or in model peptides are reported to be 3.65 and 4.25, respectively (Hunt, 2021), making them functionally similar residues and leaving them both largely deprotonated at physiological pH. These pK_a_s are not static, and large deviations from these values are not uncommon. For example, the active site of bacteriorhodopsin contains an aspartic acid with an experimental pK_a_ of 7.68 (Otto et al., 1989).

Unfortunately, protein structure elucidation by X-ray crystallography or cryogenic electron microscopy are seldom of sufficient resolution to determine locations of hydrogens, due to their extremely low electron density. X-ray crystallography detects protons only under difficult-to-achieve conditions such as resolution ∼1 Å (Woińska et al., 2016). Such resolution is not yet possible with cryo-EM. While neutron diffraction experiments can overcome this problem (O’Dell et al., 2016; Schröder and Meilleur, 2020), as it is detecting nuclei rather than electrons, experimental constraints, such as required crystal sizes, availability of neutron sources, and others, make neutron diffraction-derived structures for proteins quite rare. Multidimensional nuclear magnetic resonance methods can be applied to protein structure determination (Barrett et al., 2013), but only under certain conditions like protein size and solubility. Because NMR directly probes hydrogens, it can be used for pK_a_ determination of specific residues (Bartik et al., 1994; Schmidt et al., 2010), but this is only a probe of the residue under the NMR experimental conditions, which may differ greatly from its native physiological or solution conditions. In general, it is quite difficult to discern structural reasons for residue pK_a_ shifts experimentally, although this is a quite active area of computational research as many reports have been published suggesting what types of environments stabilize shifts (Isom et al., 2008; Isom et al., 2011; Bandyopadhyay et al., 2020). Interestingly, experimental methodologies such as NMR perform well in determining pK_a_s for surface ionizable residues but are less applicable to buried residues (Fitch et al., 2002).

Much of the effort to study protonation of ionizable residues via computational means has focused on predicting their pK_a_s by understanding the effects of other residues in the local environment. Li et al. developed a method, known as PROPKA, to empirically calculate pK_a_ values impacted by nearby residues (Li et al., 2005). In this model, hydrogen bonding to aspartates and glutamates stabilizes their deprotonated forms and lowers their pK_a_s. Spassov and Yan (2008) utilized CHARMM (Brooks et al., 1983) to develop a molecular dynamics-based approach to predict pK_a_ values of titratable groups. Several factors of 3D protein structure determination—and the resulting structural model—can compromise such predictions, e.g., uncertainties in sidechain conformations if the collected data resolution is too low (Miao and Cao, 2016).

### Computational Titration

Our lab has also previously examined this problem using our in-house force field HINT (Hydropathic INTeractions) (Kellogg et al., 1991; Kellogg and Abraham, 2000; Sarkar and Kellogg, 2010) that, briefly, exploits experimental libraries of data for atomistic partial logP_o/w_ values of small molecules and residues to account for enthalpic, entropic, and solvation contributions to free energy and score protein-ligand, protein-protein, protein-nucleotide, etc. interactions. In one study, HINT was used to predict the degree of protonation of ligand-active site interactions of neuraminidase-inhibitor complexes using a method that we termed “computational titration” (Fornabaio et al., 2003). By scoring all potential models, i.e., where the number of protons attached to ionizable residues and ligand functional groups were exhaustively enumerated, lower energy models were identified. Since proton positions are not unambiguously known from experiment, we term all such models “isocrystallographic” in that all would fit the available electron density envelope. In another report, HINT modeled the protonation state of a peptide inhibitor–HIV-1 protease complex with pH-dependent interaction scores that paralleled experimental pH-dependent binding data (Spyrakis et al., 2004).

Clearly, the presence or absence of protic hydrogens on these residue types within a protein will impact the interactions that these residues make, and in turn the protein’s 3D structure. For example, the interaction between two aspartates is radically different if one of the pair is protonated and the proton is oriented to form a hydrogen bond between them. Evaluating and understanding these phenomena is part of our long-term goal of building a new paradigm for protein structure elucidation and prediction.

### Three-Dimensional Interaction Homology

Since the dawn of protein structure elucidation, our understanding of the roles and contributions of interatomic interactions between protein residues toward biomolecular structural organization has evolved dramatically. Each of the 20 amino acid residues, regardless of how many unique protein structures they compose, is likely to situate itself within a limited set of environments with a unique system of interactions of varying magnitude, type, and loci. Our model describes four classes of interactions: favorable polar (e.g., hydrogen bond, acid-base), unfavorable polar (acid-acid, base-base, repulsive Coulombic), favorable hydrophobic (hydrophobic-hydrophobic, hydrophobic packing, π-π stacking) and unfavorable hydrophobic (hydrophobic-polar, desolvation).

Importantly, interactions with the environment of each constituent residue of a protein contributes in some part toward its rotameric structure and the protein’s overall secondary, tertiary, and quaternary structure. Our hypothesis is that each residue has a “hydropathic valence” that must somehow be satiated by nearby interacting groups. Hydrophobic residues such as phenylalanine and leucine, by interacting with other hydrophobic groups, pack together to avoid water, while polar residues, such as the three of this study, favor environments where they can engage in polar interactions, e.g., hydrogen bonding, with other residues or water. Thus, obviously, 3D protein structure is not driven by “primary” structure, but by the hydropathic interactions that each residue must make based on its type and sidechain and backbone conformations.

In our first report to address this concept, we calculated 3D hydropathic interaction maps to visualize and probe all possible environments of tyrosine (TYR) using a dataset of ∼30,000 residues. Our analysis organized all of our TYR residues into 262 unique, backbone-dependent environments, each with a unique map encoding the specific interactions made by the residue in that environment (Ahmed et al., 2015). A similar analysis with over 57,000 alanine (ALA) residues, separately calculating backbone-environment and sidechain-environment maps, yielded 136 and 150 backbone- and sidechain-dependent maps, respectively, despite ALA’s simplicity. We concluded that ALA’s mapped environments are a new and insightful form of structural motif (Ahmed et al., 2019). Recently, in our report on phenylalanine, tryptophan, and tyrosine, we showed that the subtle effects of π-π and π-cation interactions are encoded in their 3D hydropathic interaction maps (AL Mughram et al., 2021). In a report on serine and cysteine we highlight the major structural features—similarities and differences—between these two isosteric residues (Catalano et al., 2021). Importantly, our analyses describe residues by cataloguing their environments in terms of interactions and not identity. A water molecule oriented for a residue can play the same “acidic” role as a TYR–OH or a LYS–NH_3_^+^ to satisfy its hydropathic valence. Protein structure is driven by the set of these hydropathic interactions for each residue.

In the current report, we focus our attention on the hydropathic environments of aspartic acid, glutamic acid and histidine, three residue types considered to be “ionizable”, extracted from the same relatively large dataset of X-ray crystallographic protein structures. Following the same logic used in our previous work, we believe that, not only are each of these residues likely to make their own unique sets of interactions that can be clustered, but their environments also determine each residue’s unique ionization state. Thus, using our scoring methods, we have simulated titration of thousands of each of these ionizable residue types to model their protonation in available crystal structures by computationally varying pH. We have generated interaction maps similar to those in our reports on tyrosine, alanine, phenylalanine, and tryptophan, but with each possessing an individually optimized protonation state. The role of sidechain buriedness was examined using a calculated solvent-accessible surface area for each of the extracted residues. Further, we show that each residue’s backbone conformation plays a significant role in determining these protonation states. With these, we can directly predict a specific residue’s ionization state, explore the effects of varying pH, i.e., tuning, on their hydropathic environments, and collect 3D interaction-similar residue environments by clustering. Moreover, we highlight the most common environments that contribute to one state or another, but more importantly we have developed a basis set of 3D backbone-dependent residue interaction profiles for these three residues that are pieces of the protein structure analysis and prediction puzzle.

## Materials and Methods

### Dataset

From a collection of 2,703 randomly selected proteins from the RCSB Protein Data Bank, using only structures containing no ligand or cofactor, we extracted all ASP, GLU, and HIS residues from each structure, excluding N- and C-terminal residues. For these structures, we have previously described our selection criteria (Ahmed et al., 2015). Our intention was to abide by random population-based sampling of a variety of primary, secondary, and tertiary structures, thus not excluding proteins with similar or identical sequences. We believe the size of our dataset should exhaust all unique residue environments of HIS, ASP, and GLU. Hydrogen atoms were added to all heavy atoms of all structures based on their hybridization states. Positions of these atoms underwent conjugate gradient minimizations.

### Alignment Calculations

We overlayed an 8 by 8 “chessboard” on the standard Ramachandran plot, where each “chess square” has dimensions of 45° by 45° in ϕ (phi)–ψ (psi) space. The grid of the board was shifted by −20° and −25° in the ϕ and ψ directions, respectively, to enclose higher-density regions of the plot within single squares. The ϕ, ψ, and χ angles were all calculated for every residue in our dataset, and each residue was binned into their proper chess square based on its respective ϕ and ψ angles. All residues in each chess square were further divided by their χ_1_ angles into three parse groups: group “0.60”, (0° ≤ χ_1_ < 120°), group “0.180” (120° ≤ χ_1_ < 240°), and group “0.300” (240° ≤ χ_1_ < 360°). In the case of GLU, residues were still further parsed by their χ_2_ angles, yielding a total of nine parses for this residue. Supplementary Table S1 contains all information for each residue of each type in our dataset, including their chess squares, parses, PDB IDs, ϕ, ψ and ω torsion angles and atom numbers for the backbone atoms and CB of each residue.

A single model residue of each type was constructed at the center of each chess square with characteristic ϕ and ψ angles for that centroid. The CA of the peptide backbone was placed at the origin with the CA-CB oriented along the z-axis and the CA-HA bond oriented into the -y, -z quadrant of the yz-plane. All residues of each type were aligned to this model, and rotation and translation matrices were calculated by least-squares fitting of the residue constituent atoms to the model. This effectively shifted coordinates of every protein structure to align the residue of interest with the centroid within a common frame and ensures that all calculated maps and environments are attributable to a residue’s interactions and not misalignments in backbone structure. The average root-mean square distances (RMSDs) for superimpositions of backbone atoms in each chess square are close to 0.15 Å, indicating that errors arising from aligning residue backbones to the centroid model (based on the CA-CB bond) are minimal.

### HINT Scoring Function

The HINT forcefield (Kellogg et al., 1991; Kellogg and Abraham, 2000; Sarkar and Kellogg, 2010) was used for all scoring of interactions between protein atoms. HINT relies on atom-focused parameters, namely the hydrophobic atom constant (*a*_1_) and a value for solvent-accessible surface area (SASA, *S*_*i*_) for atom *i*. Generally speaking, *a*_*i*_ > 0 for hydrophobic atoms and *a*_*i*_ < 0 for polar atoms.

*S*_*i*_ is greater for more solvent-exposed external atoms. The interaction score between atoms *i* and *j* is calculated by:

$$b_{ij}=a_{i}S_{i}a_{j}S_{j}T_{ij}\;e^{-r}+L_{ij},$$

where r is the distance in angstroms between atoms *i* and *j*. *T*_*ij*_ is equivalent to −1, 0, or 1 to account for acidic, basic, etc. character of atoms involved and assign the proper sign to the interaction score. Finally, L_*ij*_ implements the Lennard-Jones potential function (Kellogg et al., 1991). *b*_*ij*_ > 0 for favorable interactions, such as Lewis acid-base and hydrophobic-hydrophobic interactions, while *b*_*ij*_ < 0 for unfavorable interactions, including hydrophobic-polar or Lewis base-base interactions.

### Computational Titration of Ionizable Residues

To determine the optimal ionization state of each studied residue, we adapted an algorithm that we reported previously for improving protein-ligand models for scoring (Kellogg et al., 1991; Kellogg et al., 2004; Sarkar and Kellogg, 2010). Our algorithm scores all possible ionization states of a model residue with other residues in its environment. Here, we optimized the ionization states of residues by first calculating the normal (environment-free) cost for ionizations of these residues using published data (ASP, pK_a_ = 3.65; GLU, pK_a_ = 4.25; HIS, pK_a1_ = 6.00, pK_a2_ = 14.44) (George et al., 1964) and applying the Henderson-Hasselbalch equation. For ASP, at pH 7, log [(CO_2_^–^]/(CO_2_H)] = 3.35, which is an equilibrium constant that can be converted to a ΔG of 4.57 kcal mol^−1^. Using the previously reported relation that −1 kcal mol^−1^ ≈ 500 HINT score units, the energy cost in HINT score units for protonating aspartate at pH 7, in the absence of local pH effects is 2,295. Table 1 summarizes these energy costs.

**TABLE 1:** Energy costs in HINT scores for computational titration of aspartic acid, glutamic acid and histidine at various pH values.

	pK_a_	pH 4	pH 5	pH 6	pH 7	pH 8	pH 9	pH 10
Aspartic Acid^a^	3.65	240	925	1,610	2,295	2,980	3,665	4,350
Glutamic Acid^a^	4.25	−171	514	1,199	1884	2,569	3,254	3,939
Histidine K_a1_^b^	6.00	−1,370	−685	0	685	1,370	2055	2,740
Histidine K_a2_^c^	14.44	7,151	6,466	5,781	5,096	4,411	3,726	3,041

The second term, calculated for each residue in varying protonation states, also as a HINT score, measures the effects of the local environment around the residue. This assessment of the environment scores the interactions of the residue in question with those nearby, in each accessible protonation state. These scores are summed together with the appropriate values in Table 1 to determine the best scoring, and therefore most likely, protonation state of the residue. For ASP and GLU, we examined the ionized (carboxylate, CO_2_^–^) and neutral states with protonation at each oxygen atom (OD1/OE1 and OD2/OE2). For the latter, the -C-C-O-H dihedral angles were exhaustively optimized for ideal hydrogen bonding to surrounding residues. For HIS, four potential ionization states exist: 1) protonation at both ND1 and NE2 (HIS^+^), 2) protonation at only ND1 (HIS-δ), 3) protonation at only NE2 (HIS-ε) and 4) deprotonated (HIS^−^), the last of which is reported to be exceedingly rare. Since the entire imidazole ring of HIS can be flipped, the potential cases for this residue are doubled to eight (*vide infra*). If the HINT score was 50 or more (∼0.1 kcal mol^−1^) than the starting case, the residue’s molecular model was replaced with the (protonated or deprotonated) trial model for that case. All further calculations at that pH were performed with the resulting optimized residue structure and coordinates.

### pK_a_ Calculations

We identified 94 residues with experimental pK_a_ values in the PKAD database (Pahari et al., 2019) that were also present in our dataset and compared our predicted pK_a_ values for those to their experimental values. Using the technique described above, we calculated individual pK_a_ values for these residues and compared them with those in the PKAD database. Calculation of a residue’s protonation state was performed within a range from 1 to 14 in increments of a quarter of a pH unit. We treated the two points representing the protonation transition state as part of a linear regression and solved for the “equivalence point” between them.

### HINT Basis Interaction Maps

Each residue with its CA-CB bond along the z-axis, was placed within a three-dimensional box large enough to accommodate the structure of a residue, plus an additional 5Å on each dimension. These boxes, based on residue type, are as follows: ASP, –8.5 Å ≤ x ≤ 8.5 Å; –8.5 Å ≤ y ≤ 8.5 Å; –7.5 Å ≤ z ≤ 9.5 Å, (42,875 points, 4,913 Å^3^); GLU, –8.5 Å ≤ x ≤ 8.5 Å; –8.5 Å ≤ y ≤ 8.5 Å; –7.5 Å ≤ z ≤ 10.5 Å, (45,325 points, 5,202 Å^3^); and HIS, –10.0 Å ≤ x ≤ 10.0 Å; –10.0 Å ≤ y ≤ 10.0 Å; –7.5 Å ≤ z ≤ 9.5 Å, (58,835 points, 6,800 Å^3^); all with a point spacing of 0.5 Å. As described previously (Ahmed et al., 2015), HINT was used to calculate an interaction grid representing the 3D interaction space surrounding a residue of interest. In short, these maps interpret sums of pairwise HINT scores (Kellogg et al., 1991; Kellogg and Abraham, 2000; Sarkar and Kellogg, 2010) into 3D map objects indicating position, intensity, and type of interaction between atoms of the residue and those close in proximity. Each grid point for a map was calculated, according to:

$$\rho_{xyz}=\sum b_{ij}\exp\left\{-\left[{\left(x-x_{ij}\right)}^{2}+{\left(y-y_{ij}\right)}^{2}+{\left(z-z_{ij}\right)}^{2}\right]/\sigma \right\},$$

where *ρ*_*xyz*_ is the map interaction score at coordinates (*x*, *y*, *z*), *x*_*i*j_, *y*_*ij*_ and *z*_*ij*_ are coordinates of the midpoint of the vector between atoms *i* and *j*, and σ is the width of the Gaussian map peak, 0.5 for our purposes (Ahmed et al., 2015). Map data were calculated for sidechain atoms of all ASP, GLU, and HIS residues with individual maps for the four interaction classes: favorable/unfavorable polar and favorable/unfavorable hydrophobic.

### Calculation of Map-Map Correlation Metrics

Comparison of two maps, **m** and **n**, are based on:

$$\mathrm{if}\left|Gt\right|/F>1.0,\;A_{t}=\left(G_{t}/\left|G_{t}\right|\right)\log_{10}\left(\left|G_{t}\right|/F\right);\;\mathrm{else},\;A_{t}=0,$$

where each raw map data point (*G*_*t*_, for point at index *t*) is transformed to log_10_ space and normalized with a predefined floor value, F = 1.0. Similarity between maps **m** and **n**, defined as *D* (**m**,**n**) is calculated based on previous methods (Ahmed et al., 2015):

$$D\left(m,n\right)=\sum \left\{1-{\left(\left|A_{t}\left(m\right)-A_{t}\left(n\right)\right|\right)}^{2}/\left[\left(\left|A_{t}\left(m\right)\right|+\left|A_{t}\left(n\right)\right|\right)\cdot \left({\left|A\left(m\right)\right|}_{\max}+{\left|A_{t}\left(n\right)\right|}_{\max}\right)\right]\right\}.$$

In this metric, *A*_*t*_(**m**) and *A*_*t*_(**n**) are map values for the same grid points in maps **m** and **n**, respectively, and |*A*|_max_ is the absolute max value of the grid points in **m** and **n**. Our map boxes are designed to accommodate all possible residue environments and usually contain a majority (>60%) of zero-valued points. To mitigate the issue that all map pairs would appear similar, only points where |*A*_*t*_(**m**)| ≥ 8 |*A*(**m**)_stddev_| or |*A*_*t*_(**n**)| ≥ 8 |*A*(**n**)_stddev_| (*A*_stddev_ is the standard deviation of the average value of all points in the map) in calculating *D* (**m**,**n**) (Ahmed et al., 2015) were considered.

*D* (**m**,**n**) should normally range from 0 to 1, where 1 indicates identical maps; realistically, *D* (**m**,**n**) = 0 cannot exist, as it would signify completely overlapping maps with opposite signs. Neither will *D* (**m**,**n**) = 0.5 exist, as it would require completely non-overlapping maps. Typically, the minimum *D* thus falls between 0.6 and 0.7. To calculate the overall similarity (***D***_*all*_) between two like residue maps **m** and **n**, one composite metric was calculated from four metrics containing data for the map quartet described above [hydro (+), hydro (−), polar (+), and polar (−), which are favorable and unfavorable hydrophobic (e.g. hydrophobic-polar) contributions, and favorable and unfavorable polar contributions to each map, respectively]. Here, ***D*** (**m**,**n**)_*all*_ = { 4[*D* (**m**,**n**)_hydro(+)_] + 2[*D* (**m**,**n**)_hydro(–)_] + [*D* (**m**,**n**)_polar(+)_] + [*D* (**m**,**n**)_polar(–)_] }/8.

The favorable and unfavorable hydrophobic interactions were scaled by 4 and 2, respectively; these two terms are more subtle, diverse and potentially information-rich, than those driven by electrostatic, particularly ionic, interactions.

Also, to reduce the computational burden, we applied a first-pass similarity filter (Ahmed et al., 2015) to our matrix calculations to remove certain residues from further consideration because many maps are highly similar as they share highly similar environments, and thus can be removed to avoid redundancy. This significantly scales down our pool of calculations, which is significant as several steps scale more or less as n^2^.

As described previously (Ahmed et al., 2015), all above calculations were performed with in-house-written programs that exploit the inherent parallelism of our methods with GPUs, specifically used to calculate maps and similarity matrices.

### Clustering and Validation

We utilized the freely available R programming language and environment (R Core Team, 2013) to perform our clustering analysis on the pairwise map similarity matrices calculated above. We determined (Ahmed et al., 2015) that for our purposes, out of a number of different clustering methods, the k-means method was most reliable. Through the experience of our previous reports (Ahmed et al., 2015; Ahmed et al., 2019) and preliminary studies here, we opted to set a uniform maximum number of clusters of 12 for each chess square-parse combination. This allows for significant map diversity and facilitates inter-chess square/inter-residue comparisons. Most ***chess squares/parses***, however, had fewer than 12 clusters in their optimal solutions. Additionally, k-means clustering will not form singleton clusters, i.e., with a single member. However, while this is fairly rare (∼5%), these maps could be interesting, so our protocols are designed to optionally recover them by reconstructing the cluster solutions with the missing singletons. Any chess square-parse with four or fewer maps was not clustered, but, instead, averaged to create what is, effectively, a 1-cluster case.

### Average Map, RMSD, and Solvent-Accessible Surface Area Calculations

Careful consideration must be given to calculation of average maps. First, to avoid what we have described as “brown mapping” (Ahmed et al., 2015), only maps sharing high similarity should be combined. Second, the average maps are calculated by Gaussian weighting (*w*) the contribution of each map with respect to its Euclidean distance from the cluster centroid, given by:

$$w=\exp\left[-\left(d^{2}/\sigma^{2}\right)\right],$$

where *d* is the map’s distance from the centroid and σ = *d*_max_/8, which is the average of all maximum distances across all clusters in the chess square. This weighting ensures that maps closer to the centroid contribute more significantly to the average map of the cluster, whereas taking a flat average of all map data would overweight the importance of maps further from the centroid. While a formal definition exists for “exemplar” in affinity propagation clustering, for our purposes, it represents the residue datum closest to the centroid of each cluster output by the k-means algorithm.

RMSDs (root-mean square distances) for each residue type were calculated by weighted averaging, as above, all atomic positions from all residues in a cluster to construct one average residue structure. For each non-hydrogen atom, an RMSD was calculated from the average structure, and then all atomic values were averaged to obtain the reported RMSD for the cluster.

We calculated SASAs for all residue sidechains using the GETAREA algorithm (Fraczkiewicz and Braun, 1998) and its default settings. The protein coordinates in PDB files were submitted as input. Also from GETAREA’s “In/Out” parameter, we created a new metric “*f*_*outside*_” to represent the buriedness of the set of residues in a cluster, parse, chess square, etc. by recasting “In” as 0.0, “Out” as 1.0 and “indeterminant” as 0.5, and averaging the set.

## Results and Discussion

### Dataset: Binning and Parsing Residues

**FIGURE 1:**
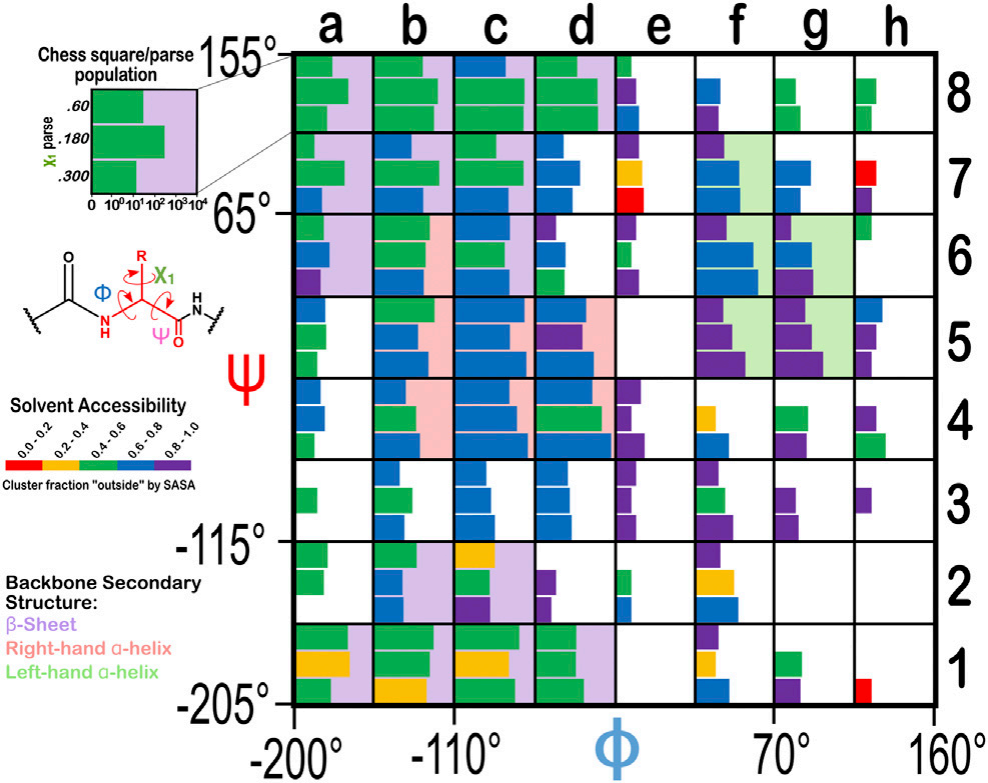
Ramachandran chessboard displaying the chess square/parse population for aspartic acid. The Ramachandran ϕ vs. ψ plot is rendered into 64 45° by 45° (π/4 by π/4) chess squares. The (χ_1_) parse populations for ASP are represented in log_10_ scale with the colored bars. Their colors reflect the average weighted fraction outside or solvent exposed, i.e., “*f*_*outside*_”, a measure of solvent accessibility (see text for definition). The ϕ vs. ψ regions associated with β-pleat, α-helix, and left-hand α-helix secondary structure motifs are shaded in light purple, light orange and light green chess squares, respectively.

**FIGURE 2:**
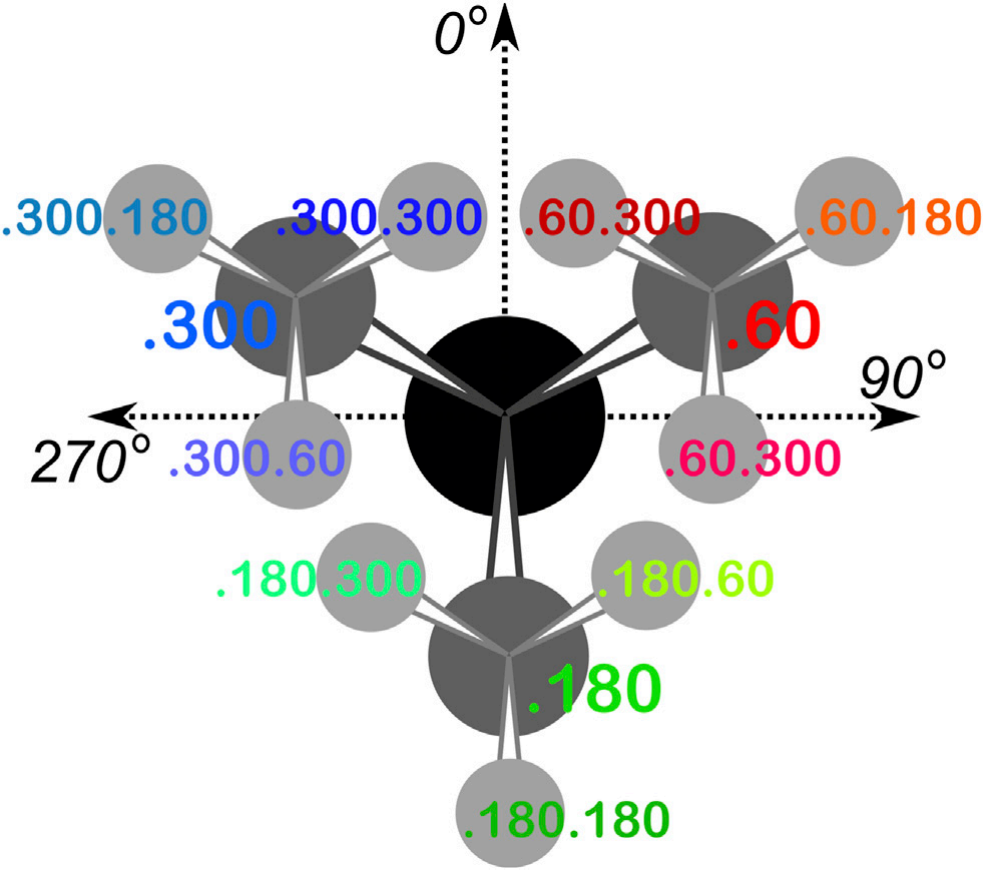
The χ_1_ and χ_2_ rotamer parses. CB (black) has three χ_1_ rotamers (dark gray, CG): 0.60, 0.180 and 0.300. Each of those, for GLU, has three χ_2_ rotamers (light gray, CD), as shown.

From the dataset of 2,703 protein structures described in Methods, we extracted 42,713 ASPs, 49,306 GLUs, and 15,276 HISs, all of which were non-terminal residues. An 8 by 8 chessboard was overlaid on a standard Ramachandran plot (Ramachandran et al., 1963), such that each grid square has dimensions of 45° by 45° in ϕ–ψ space and the extents of the board are shifted slightly to contain regions of high residue population density in single squares (Figure 1), named as ***a1*** through ***h8***. We binned residues into each square by their backbone ϕ and ψ angles and further parsed them by their χ_1_ angles into three groups corresponding to those normally observed in rotamer libraries (Shapovalov and Dunbrack, 2011): a group averaging ∼60°, a group averaging ∼180°, and a group averaging ∼300° from here on referred to as the “0.60”, “0.180”, and “0.300” parses. In the case of GLU, residues were still further parsed by their χ_2_ angles, yielding a total of nine parses for this residue: “0.60.60”, “0.60.180”, “0.60.300”, “0.180.60”, “0.180.180”, “0.180.300”, “0.300.60”, “0.300.180” and “0.300.300” (Figure 2). We showed previously (Ahmed et al., 2015) that map-based clustering was able to easily identify this (χ_1_, χ_2_) low level of detail, except for surface-exposed residues that show few interactions with anything apart from solvent. However, even a few such failures were problematical in calculating average maps and residue coordinates. Furthermore, parsing of the chess square members into χ bins increased computational efficiency. (Many calculations scale as n^2^: 3 × (n/3)^2^ < n^2^). The additional χ_2_ parse for GLU further reduced the computations and made the ASP and GLU data more comparable, i.e., the (unparsed) remainder of their sidechains is the same –C–COOH fragment.

Throughout this work, chess square names will be given in bold italics, e.g., a1, b4, etc. The χ_1_ parses for ASP and HIS will be denoted by the suffixes 0.60, 0.180 and 0.300 and the χ_1_/χ_2_ parses for GLU will be denoted by the suffixes 0.60.60, 0.60.180, 0.60.300, etc.

The occupancies of the chess square/parses range from 0 to 6,215 (***d4.300***) for aspartate, to 4,563 (***d4.300.180***) for glutamate, and to 1,504 (***d4.180***) for histidine. For aspartate, 44 (of 64) chess squares contain 10 or more residues, and 159 chess squares/parses (of 192) are occupied at all. These metrics are 40/64 and 356/576 for glutamate and 32/64 and 120/192 for histidine. Supplementary Table S1 provides occupancies in the Ramachandran chessboards for these three residues. To simplify nomenclature in this article, we are using a numerical scheme wherein the sequential number of that residue in its chess square/parse is its name. Thus, histidine 100 in chess square ***a1.60*** is the 100th histidine contained within that chess square/parse combination, as tabulated in Supplementary Table S2, wherein the specific actual PDB ID, chain, residue name, etc. for each datum in this study can be found. Clusters (*vide infra*) will be named for the residue closest to its centroid or exemplar and will be given in bold numerals.

The Ramachandran plot generally contains four regions associated with specific secondary structure motifs. According to our schema (Figure 1), fifteen chess squares (***a1***, ***a6***, ***a7***, ***a8***, ***b1***, ***b2***, ***b7***, ***b8***, ***c1***, ***c2***, ***c6***, ***c7***, ***c8***, ***d1*** and ***d8***) correspond to the β-pleat motif, seven chess squares (***b4***, ***b5***, ***b6***, ***c4***, ***c5***, ***d4*** and ***d5***) correspond to the right-hand α-helix motif and five chess squares (***f5***, ***f6***, ***f7***, ***g5*** and ***g6***) correspond to the left-hand α-helix motif. The remaining chess squares, some of which may contain mixtures of secondary structural motifs, account for the remaining residues.

Calculations in this study were performed for all Ramachandran chess squares, but, for brevity’s sake, we focus our discussion on a particular four, designed to sample the three major regions of the standard Ramachandran plot: ***b1***, ***c5***, ***d5*** and ***f6***. The ***c5***, ***d5*** pair allows us to compare independently-calculated map and environment data between chess squares within the same right-hand α-helix structural motif region.

### Ionization State Optimization

While our primary goal for this study is to evaluate the hydropathic environments of the ASP, GLU and HIS residue types, a key requirement was to use molecular models that are in appropriate ionization states. We were also interested in examining the effects of these ionization states on the residue environments. Also, such structures (and 3D maps) should have rational and tunable pH dependencies to enable prediction of structure, properties, and function.

**FIGURE 3:**
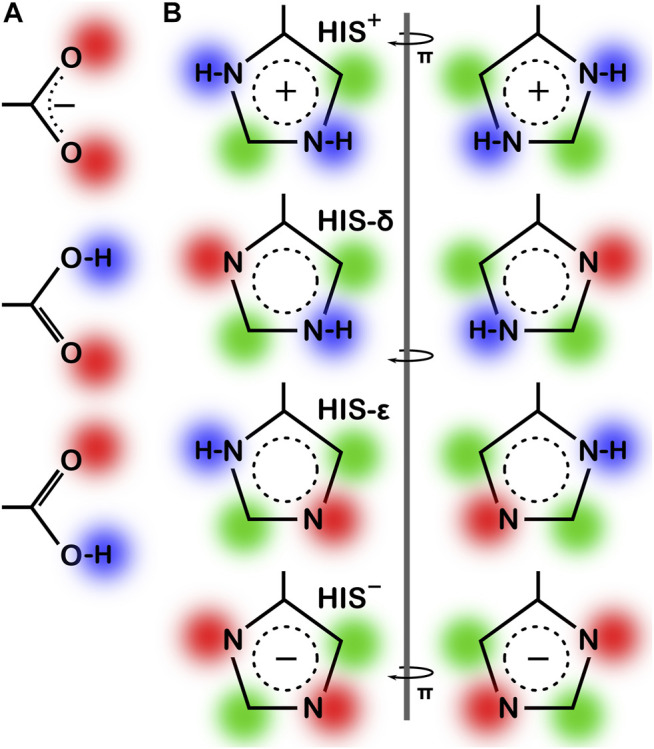
Various possibilities for ASP, GLU and HIS ionization/rotameric states. **(A)** ASP, GLU, and **(B)** HIS sidechain functional groups. Red = Lewis acid, blue = Lewis base, green = hydrophobic. Note that “ring flips” of HIS present distinct patterns for interaction.

As the local environment heavily influences protonation states of ionizable residues, we updated the computational titration algorithm that we reported earlier (Kellogg and Abraham, 2000; Fornabaio et al., 2003) to optimize the ionization state (and concomitantly the–C–O–H dihedral angle) of all residues in this study. Briefly (Methods), we calculated the HINT score between each residue and its local environment in each of its possible ionization/rotameric states (3 for ASP and GLU, 8 for HIS, Figure 3). These scores were modified by pK_a_- and pH dependent factors derived from the Henderson-Hasselbalch equation. It is important to emphasize that all these calculations were performed without changing the atomic positions of the non-hydrogen atoms—except for the π rotation about χ_2_ shown on the right side of Figure 3B. In other words, all models generated and scored are isocrystallographic. The highest-scoring model of the set generated for each residue was selected for moving forward in the study. We note an advantage here: since the positions of the heavy atoms are fixed based on their X-ray structures, calculations will likely identify the protonation model most favorable for that conformation.

#### Aspartic Acid

**FIGURE 4:**
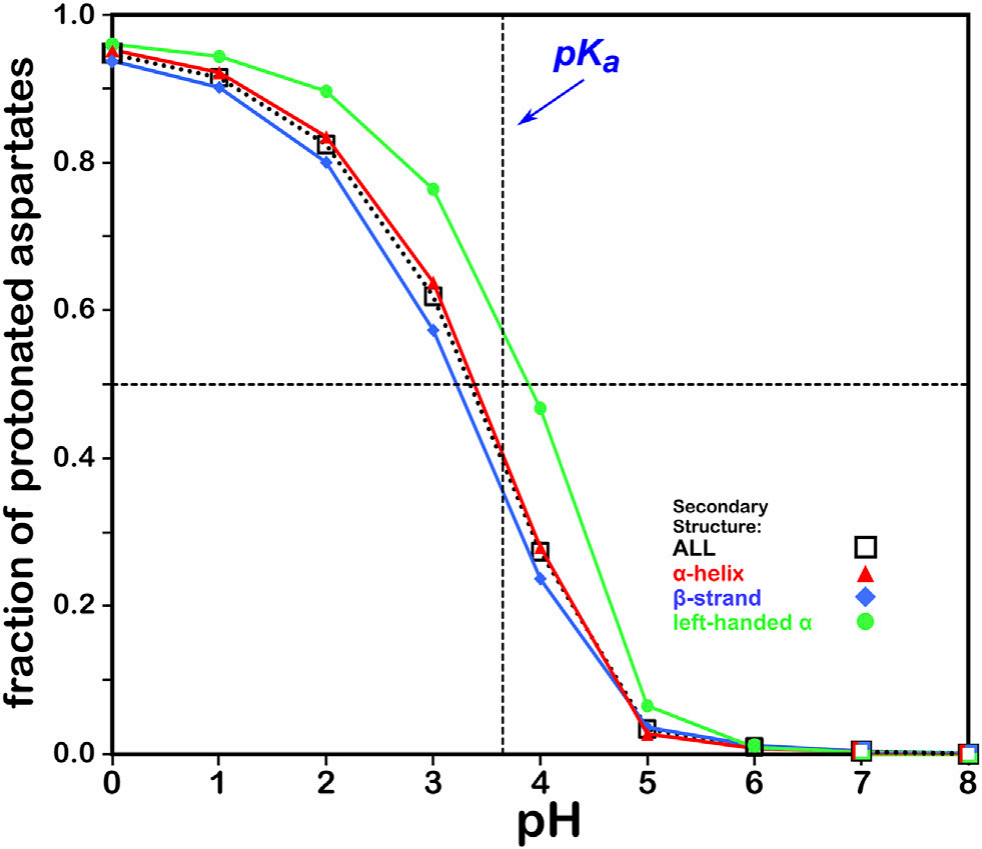
Titration curves of ASP residues by secondary structure. The native pK_a_ for aspartic acid is indicated.

We calculated the optimal structure for each studied aspartic acid at a range of pHs. For this residue, where the pK_a_ is 3.65, we determined the fraction of the nearly 43,000 residues protonated at pHs from 0 through 8. The result, which is reminscent of a titration curve, is shown in Figure 4. Our calculations yielded the total fraction of aspartic acids expected to be protonated at pHs 0 through 8 in increments of 1 with an overall titration curve centered close to the nominal ASP pK_a_ and differing, overall, by ∼0.31 pH units. Our calculations suggest that residue backbone structure has an impact on levels of protonation. Our data (*vide infra*) also suggest that differences in secondary structure have an effect on solvent accessibility: these two phenomena are intimately linked, and in fact difficult to separate. pK_a_ shifts associated with differences in solvent-accessible surface area are known, as less solvent exposure may increase the pK_a_s of acidic residues (Harms et al., 2009). Highly solvent-exposed residues are, in practice, *in vacuo* in many protein structure models so that there are no inter-residue interactions to account for. The pH in our calculations at which the aspartic acids are 50% ionized (which we are calling pH_50_) is 3.345. While this is an arbitrary value, we will use pH_50_s as set points for map calculations (see below).

#### Glutamic Acid

**FIGURE 5:**
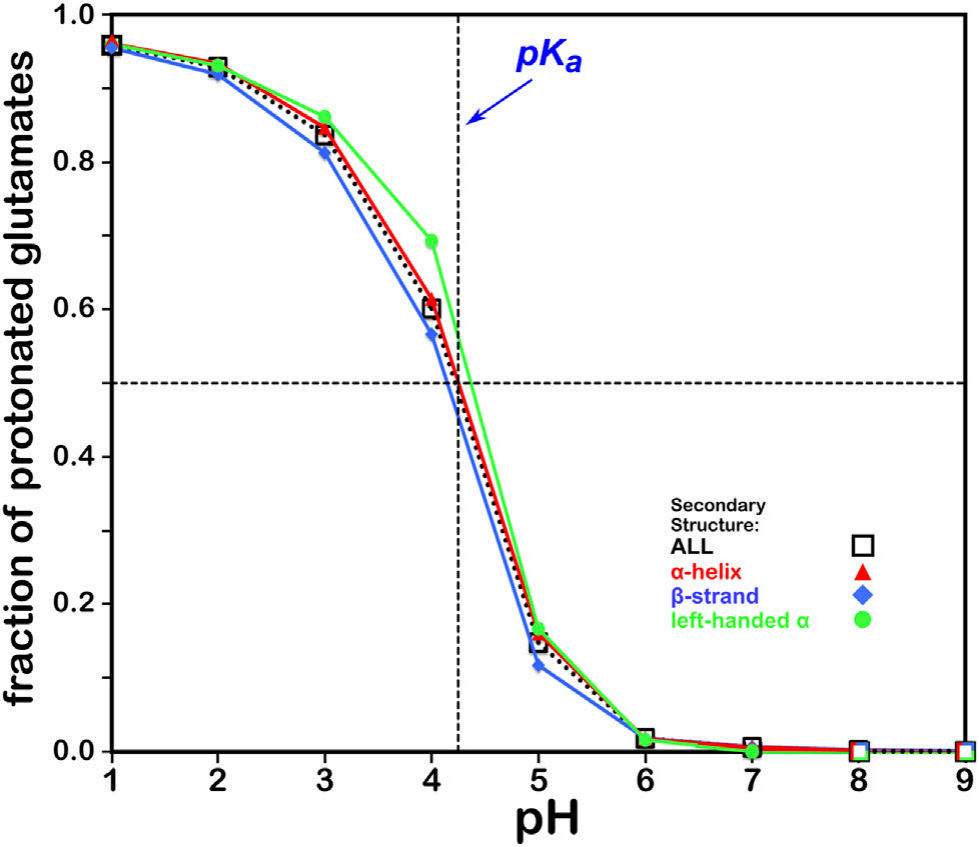
Titration curves of GLU residues by secondary structure. The native pK_a_ for glutamic acid is indicated.

The titration curves for the over 49,000 GLU residues in our study are shown in Figure 5. These look very similar to those of ASP and, in the same way, center very closely to its native experimental pK_a_. In fact, the average calculated GLU pK_a_ deviated from the experimentally-determined pK_a_ for the GLU model peptide by only ∼0.03 pH units. There is also seemingly less secondary structure dependence for these results, which is likely due to differences in solvent accessibility between ASP and GLU sidechains. pH_50_ for our glutamic acid data is 4.224.

#### Histidine

**FIGURE 6:**
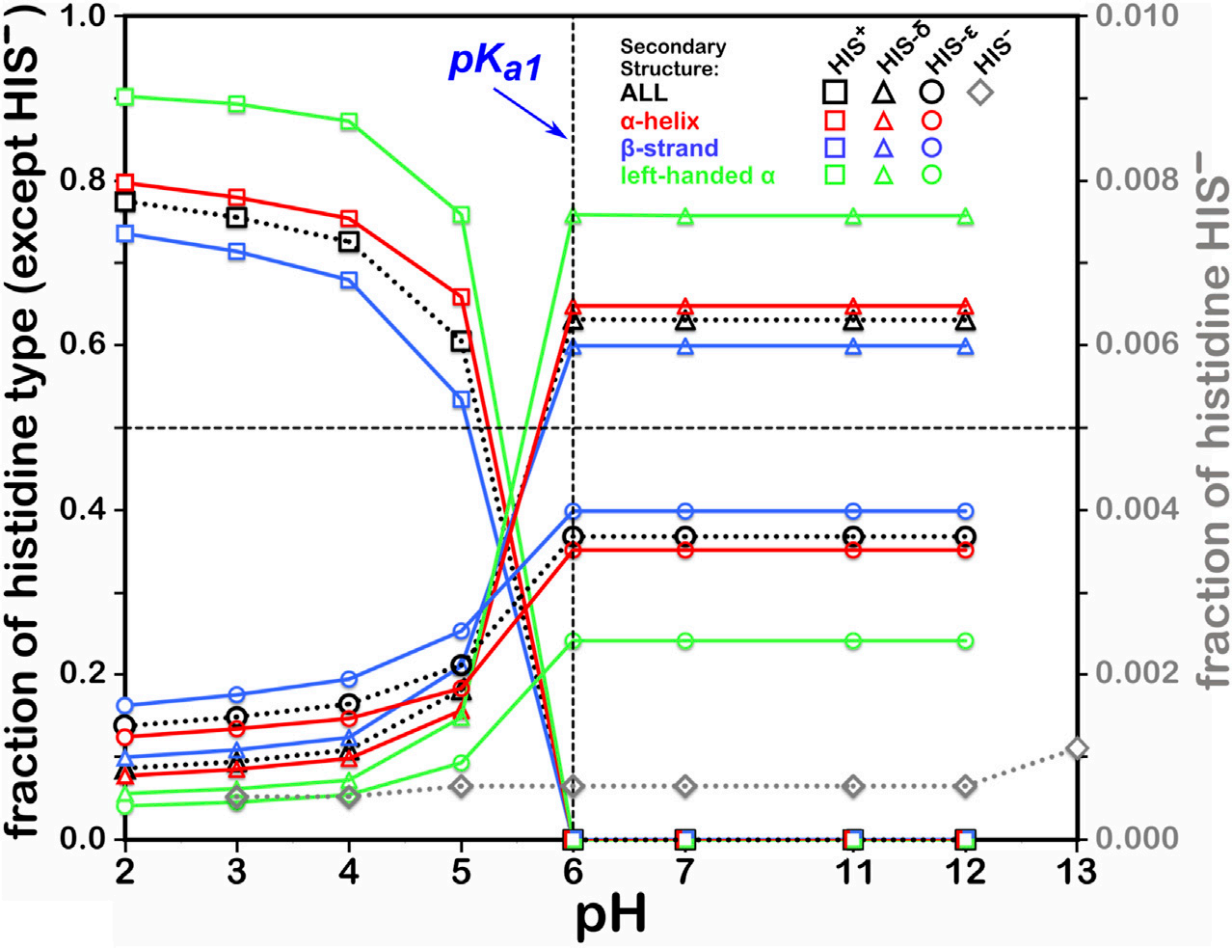
Titration curves of HIS residues by secondary structure. The native pK_a1_ for histidine is indicated. Full deprotonation of HIS to HIS^−^ is shown with data colored in gray and right-hand y-axis.

This residue type potentially has three different protonation states, resulting in four unique protonation patterns (Figure 3), compared to ASP’s and GLU’s two, and thus tells a more complicated story (Figure 6). In addition to the expected HIS to HIS^+^ protonation, HIS can be deprotonated to HIS^−^ (Ascone et al., 1997) in exceedingly rare cases, such as Cu, Zn superoxide dismutase. We simulated the titration of more than 15,000 HIS residues in our dataset together and separately by their secondary structure. According to our calculations, in the neutral state, a greater fraction of HIS residues were protonated at the ε-nitrogen in all secondary structures. However, factors contributing to protonation of HIS are much more complicated, including solvent accessibility and conformational changes, discussed later. The deviation of our calculated pH_50_ of 5.174 from the nominal HIS pK_a1_ of 6.00 is greater for HIS than those of ASP and GLU, here ∼0.83 pH units. Also interesting is that apparently only around 80% of HIS residues can even be protonated to HIS^+^, likely due to steric contraints disallowing that configuration, but for HIS in left-hand α-helix conformations, 90% can be protonated, presumably due to less structural constraint imposed by that backbone motif.

#### Summary of pH Optimization Results

Although this was a secondary goal, our predictions for residue pK_a_s are reasonable enough (Supplementary Table S3) that the molecular models upon which our 3D maps are constructed are likely to be correct, as least as snapshots of them in the dynamic biological solution. Our algorithm tends to simulate ionization for *highly solvent-exposed* residues in protonated forms (charge neutral for ASP and GLU and cationic for HIS). As noted above, there are no interacting residues and (usually) few or no explicit water molecules in the protein models for such residues to aid in the estimation, and the few interactions that are found prefer uncharged species. Our simulation of “bulk” solvent is only through the pressure applied by the external pH term in the Henderson-Hasselbalch relation. For high-level pK_a_ estimations, clearly more rigorous consideration of solvent molecules and, as Friedman (2011) showed, ions, may provide more accurate predictions of ionization states. However, on the ∼10^5^ case scale of this study, we used our more practical and accessible approach.

Interestingly, the easier to experimentally determine pK_a_s of surface residues (Fitch et al., 2002) contrasts with the easier to calculate pK_a_s of more buried residues, and there is not really a lot of experimental data available. The ionization state-optimized molecular models, which are more important for our purposes, are likely to be quite reasonable except in edge cases. The computationally more problematical highly solvent-exposed residues are fully immersed in water and are thus less participatory in protein structure. We will show below that the edge cases, themselves, are also not a significant issue because it is interactions that are assayed by the maps, and an ASP, GLU or HIS can be a donor and/or an acceptor.

### Calculation of Hydropathic Environment Maps

Based on methods in our previous reports (Ahmed et al., 2015; Ahmed et al., 2019; AL Mughram et al., 2021) we evaluated interatomic interactions using the *HINT* force field and score model (Kellogg et al., 1991; Kellogg et al., 1991; Sarkar and Kellogg, 2010), which uses two atom-centered parameters *a*_*i*_ and *S*_*i*_, the partial log *P*_*o/w*_ (for 1-octanol and water solute transfer) and a term related to solvent accessible surface area, respectively, for atom *i* to score atom-atom interactions (see Materials and Methods). We have reported previously on *HINT*’s ability to estimate changes in free energy for ligand-protein, protein-protein and other complexes in various systems, (Burnett et al., 2000; Burnett et al., 2001; Cozzini et al., 2004; Da et al., 2013), such that ∼500 *HINT* score units correlate well with a ΔΔG = −1 kcal mol^−1^.

As stated above, one of our primary hypotheses is that there is a limited set of unique 3D hydropathic interaction environments that satisfy the “valence” of a residue. These valences are based on interaction types, strengths and geometry. For example, as we showed in previous work (Ahmed et al., 2015) the phenol hydroxyl of tyrosine can make favorable polar interactions with an appropriately positioned hydrogen bond donor and/or acceptor, and it can take the form of a backbone amide, another polar sidechain, or a water molecule. In contrast, our alanine maps showed fewer unique interactions, with its methyl sidechain and no rotamers, but about four to six specific patterns appeared to be conserved (Ahmed et al., 2019). Consistent in both of these studies is that we only need to be focused on the interactions that a residue makes with its environment by class, not by the specific donor-acceptor pair or residue type identities. In other words, the *type* of interaction, its strength and location are more significant than its participants.

Maps were constructed within rectangular boxes tailored to be large enough to contain each of our three studied residue types with its interacting atoms (*Materials and Methods*). These maps are calculated to quantify the strength of the variety of interactions each residue in our dataset makes with the other atoms in its environment. Our maps categorize interactions in “quartets” of four separate types: favorable polar, unfavorable polar, favorable hydrophobic and unfavorable hydrophobic. Our previous work on tyrosine (Ahmed et al., 2015) and alanine (Ahmed et al., 2019) examined the hydropathic environments as stand-ins for structure. Here, we exploit these maps that encode extensive information concerning the structural roles of the carboxylates and sidechains of aspartate and glutamate and the dual proton acceptor-donor nature of histidine’s imidazole. Our map data further use this information to account for the environments that potentially stabilize any of these residue's ionization states, particularly in response to changes in pH.

### Evaluating the Fundamental Patterns in the Maps

To extract the information encoded in the 3D hydropathic interaction maps, we first developed a map-map similarity metric (Ahmed et al., 2015) to score two maps **m** and **n** (section *Materials and Methods*). In brief, the overall similarity (***D***_*all*_) between two like residue maps **m** and **n**, is comprised of a single scalar metric derived by the linear combination of four terms, one for each member of the map quartet contributions to each map, respectively. These scalars were loaded in square matrices, for each chess square and parse, for statistical analysis. Next, we clustered these matrices with k-means clustering within the R programming environment. As described in *Materials and Methods*, we set a maximum number of 12 clusters per chess square-parse combination; this was sufficient for capturing the diversity of residue environments while balancing computational efficiency. Supplementary Table S4 sets out the number of clusters found on a chess square-parse basis for the three residue types in this study.

### Hydropathic Interaction Maps

The objective of examining maps is to view 3D representations of the positions and magnitudes of the constellation of interactions made by residues. We expected that secondary structural differences affect the interactions a residue makes with its environment, which we enforced with the chessboard schema. Additionally, the parse inside each chess square may impact these interactions. For these reasons, we focused the analysis presented here on four particular chess squares, ***b1***, ***c5***, ***d5*** and **f6**, to survey the environments from each of the three secondary structural regions of the Ramachandran plot, as in previous reports (Ahmed et al., 2019; AL Mughram et al., 2021). We performed complete studies for all three residues at pHs 3, 5, 7, and 9 and at the pH for each residue at which half of all of that type of residue were protonated, which we named pH_50_ above. However, we only constructed visual map contours displays at each residue’s pH_50_, as we believed this pH would be best representative of the diversity of maps in protonated and deprotonated cases.

#### Aspartic Acid

Aspartic acid, by nature, is an extremely polar residue, owing to its carboxy acid sidechain. For this reason, we expected to see two things: 1) a plethora of maps indicating strong favorable and unfavorable polar interactions localized around the carboxylate end of the sidechain and 2) many clusters of maps with high solvent-accessible surface areas, due to the high presence of ASP residues on protein exteriors. Indeed, many clusters of ASP within our studied chess squares show intense positive and negative polar interactions surrounding the carboxylate, particularly in clusters with low SASA. Those maps that appear largely void of interactions are in clusters with high solvent-accessible surface area, where, as we noted above, there are no residue-protein interactions.

**FIGURE 7:**
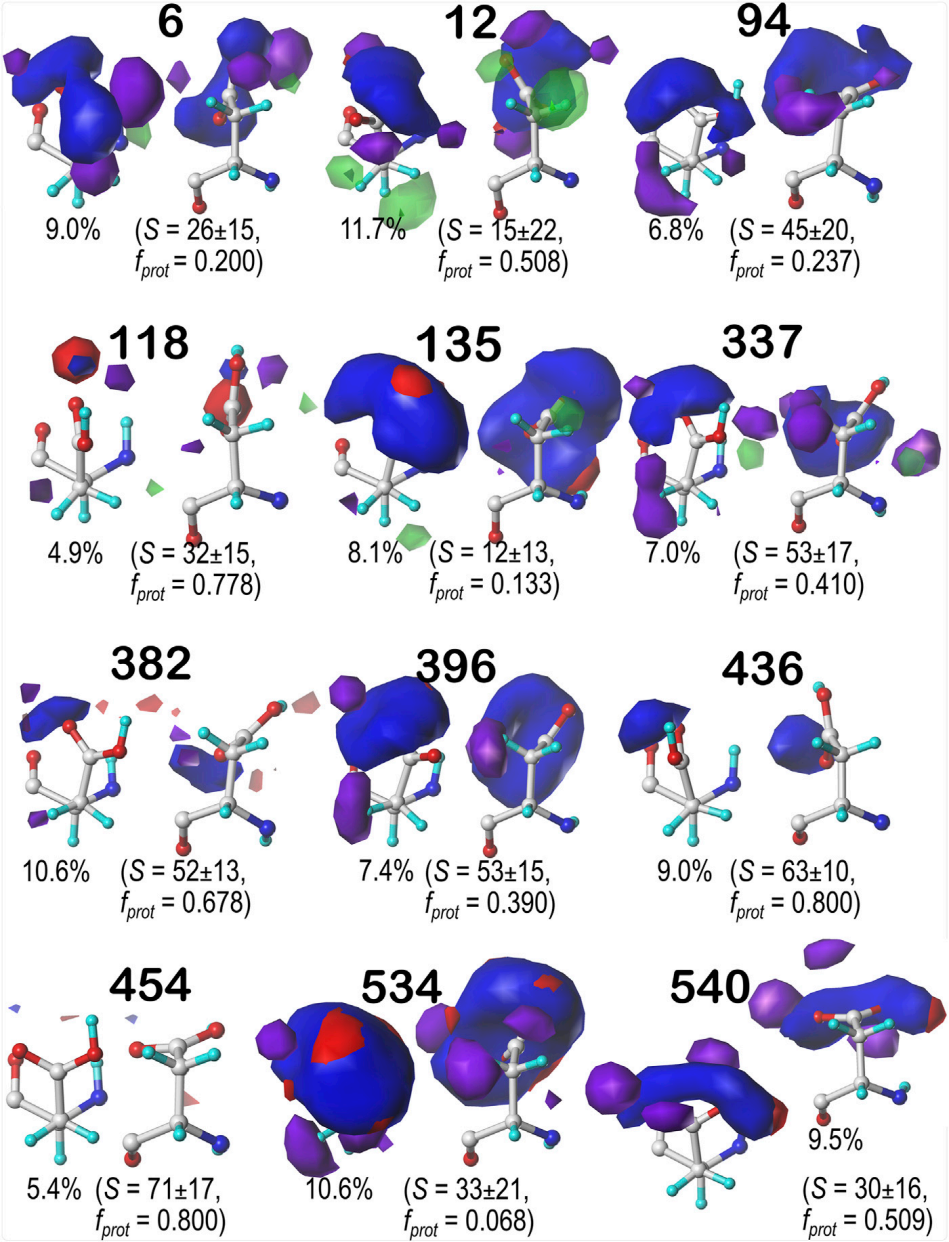
Hydropathic interaction maps displaying the Gaussian-weighted average sidechain environments of aspartic acid in the χ_1_ = 60° parse of the *b1* chess square at pH = 3.345. Two map viewpoints are given for each cluster, whose ID is given in bold. The left map in each pair is oriented such that the CA-CB z-axis bond points upward, while the right is oriented to point it out of the page. The x-axis is oriented horizontally in both. The percentage indicates the fraction of the parse represented by that cluster. *S* represents the solvent accessible surface area in Å^2^, and *f*_*prot*_ indicates the fraction of the cluster protonated at pH_50_. Blue contours indicate positive polar interactions made with the sidechain, and red indicates negative polar interactions, while green and purple indicate positive and negative hydrophobic interactions, respectively.

**FIGURE 8:**
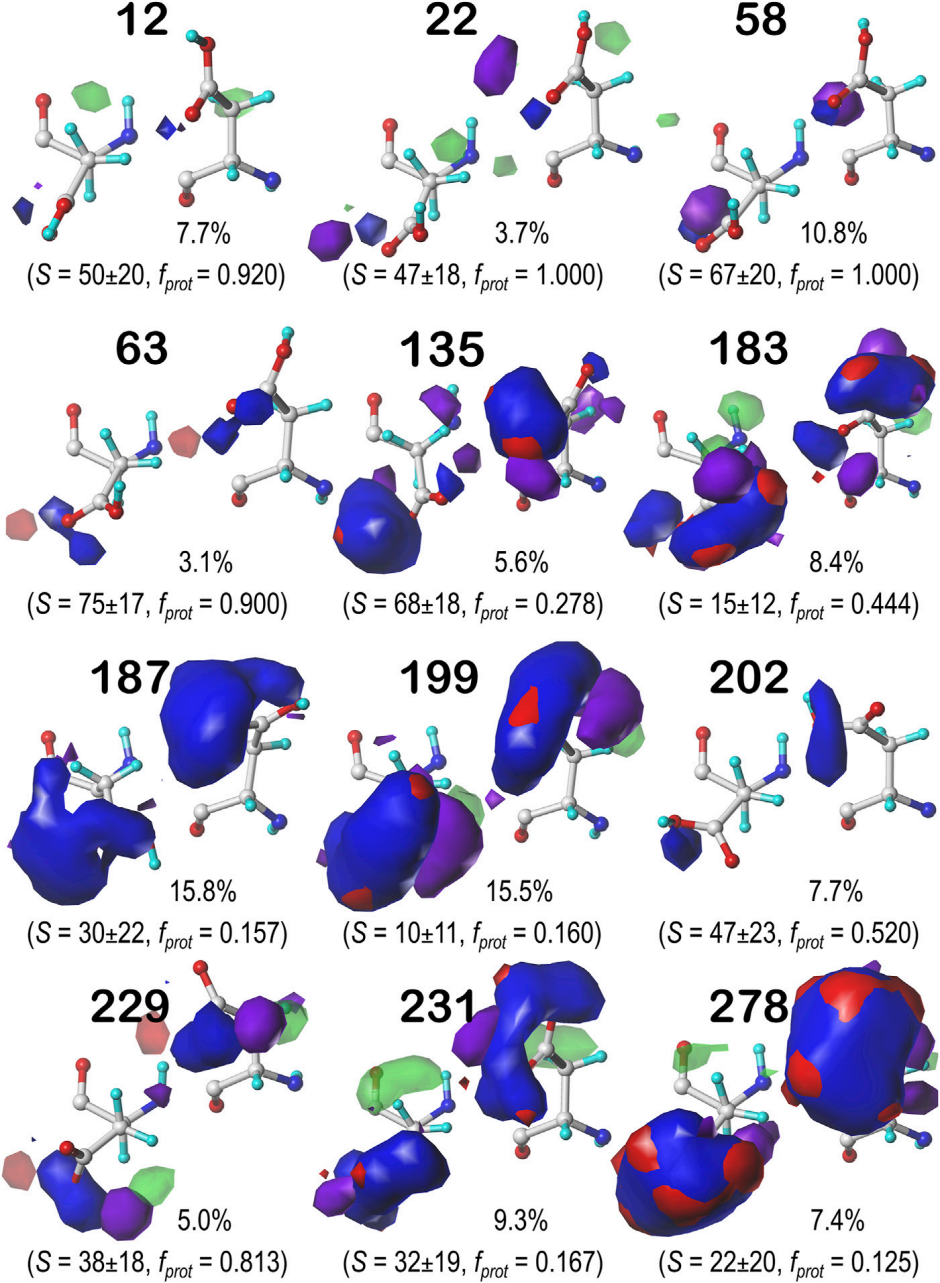
Hydropathic interaction maps displaying the Gaussian-weighted average sidechain environments of aspartic acid in the χ_1_ = 180° parse of the *b1* chess square at pH = 3.345. See caption for Figure 7.

**FIGURE 9:**
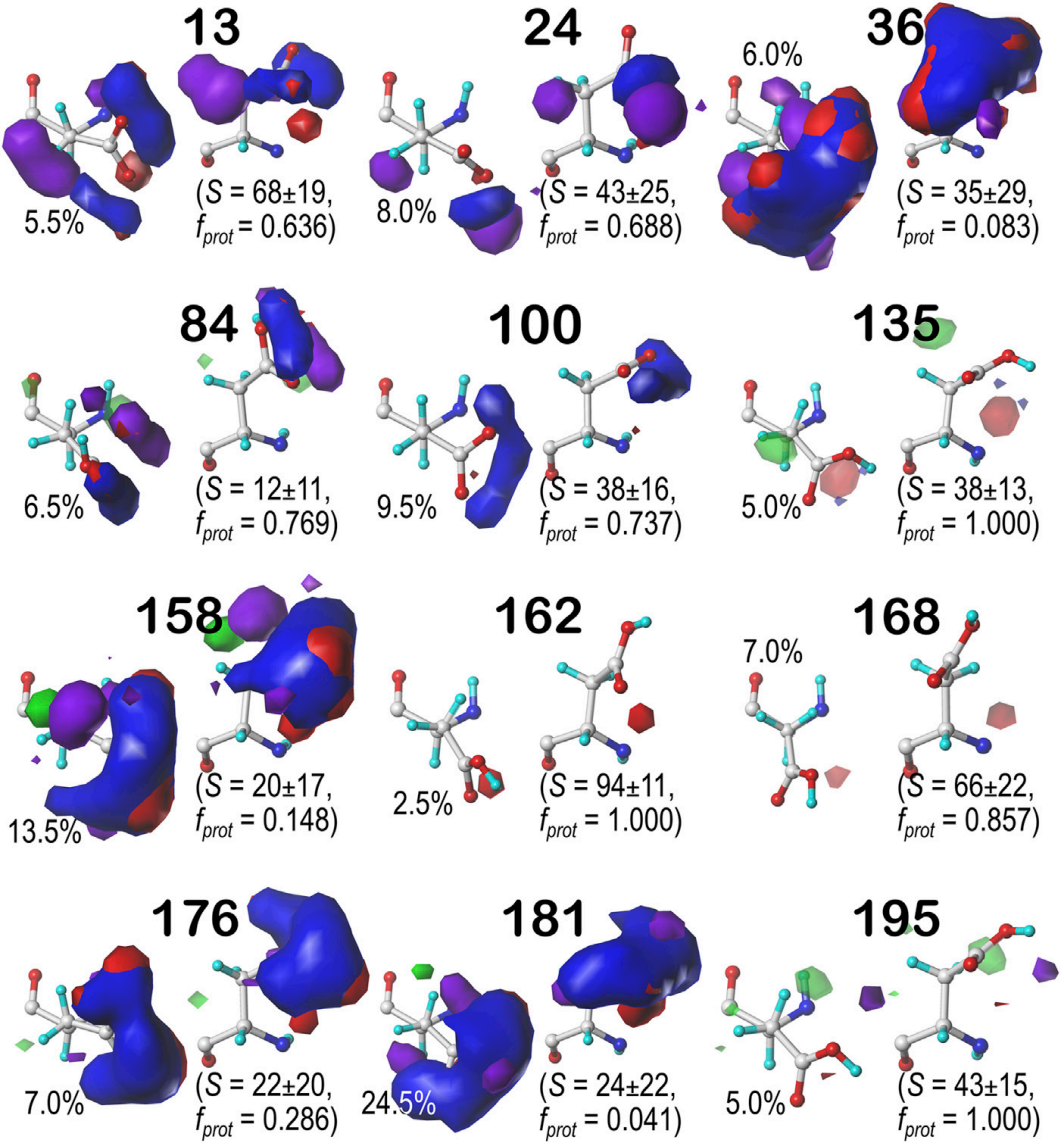
Hydropathic interaction maps displaying the Gaussian-weighted average sidechain environments of aspartic acid in the χ_1_ = 300° parse of the *b1* chess square at pH = 3.345. See caption for Figure 7.

For brevity, we are discussing in more detail ASP residues in the ***b1*** chess square, but further detail on the ***c5***, ***d5*** and ***f6*** chess square results are in Supporting Information. Aspartic acid residues in the ***b1*** chess square appear to be, comparatively, the least solvent-exposed of the four squares, yielding more robust sidechain interactions; this point is the subject of further discussion in a later section. Figures 7–9 display the contoured maps for ASP in the 60°, 180° and 300° parses of ***b1***, respectively. The percentile contribution of each cluster to the chess square/parse is listed, along with the average GETAREA (Fraczkiewicz and Braun, 1998) SASA (*S*) and the fraction of the members of that cluster that are protonated (*f*_*prot*_).

One significant point is that the displayed contours, as they represent a map, are showing *interactions*. Thus, cases where the ASP is ionized (acting as an H-bond acceptor) interacting with a donor could be indistinguishable from cases where the ASP is protonated (acting as a donor) interacting with an acceptor. Thus, it is entirely reasonable for some clusters to have a mixture of ionized and protonated ASPs, although most have *f*_*prot*_ ≤ 0.2 or *f*_*prot*_ ≥ 0.8. Most interactions shown are of the positive polar type, which is appropriate, given the role we expect ASP to serve. These are the prominent, mostly blue contours near the carboxy acid/carboxylate oxygens that signify hydrogen bonds between one or both of these atoms and their environment. Additionally, many clusters in buried environments with low SASA (<20 Å^2^) were calculated to be largely deprotonated, i.e., ASP in this environment is acting as a hydrogen bond acceptor. However, some clusters showed high degrees of protonation at pH_50_ = 3.345, such as clusters **12**, **118** and **540** in ***b1.60*** (Figure 7) and **84** in ***b1.300*** (Figure 9). Cluster **84**, in particular, showed protonation of 77% of its members with a SASA of 13 ± 12 Å^2^ at this pH.

Contour maps for the ***c5***, ***d5*** and ***f6*** chess squares show largely similar map profiles, and are presented in Supplementary Figures S1, S2 for ***c5*** parses 0.60, 0.180 and 0.300, respectively; in Supplementary Figures S4–S6 for ***d5*** parses 0.60, 0.180 and 0.300, respectively; and in Supplementary Figures S7–S9 for ***f6*** parses 0.60, 0.180 and 0.300, respectively. Further numerical data supporting these results and encompassing all chess squares is provided in Supplementary Figure S5. In summary, each map appears to be a backbone-specific representation of a unique collection of interactions made by an aspartate/aspartic acid residue. To demonstrate this, we calculated inter-cluster similarities using the previously described algorithms. The average cluster-cluster similarities *within* chess squares are: 0.799 in ***b1***, 0.795 in ***c5***, 0.791 in ***d5***, and 0.802 in ***f6*** chess squares. However, a few pairs of cluster maps in the adjacent chess squares ***c5*** and ***d5*** have similarities of >0.900: **637** (***c5.60***) and **146** (***d5.60***)*,* **57** (***c5.180***) and **70** (***d5.180***), and **217** (***c5.300***) and **58** (***d5.300***), indicating that backbone secondary structural elements may encode inherent similarities in the kinds of environments likely to surround a given residue.

#### Glutamic Acid

**FIGURE 10:**
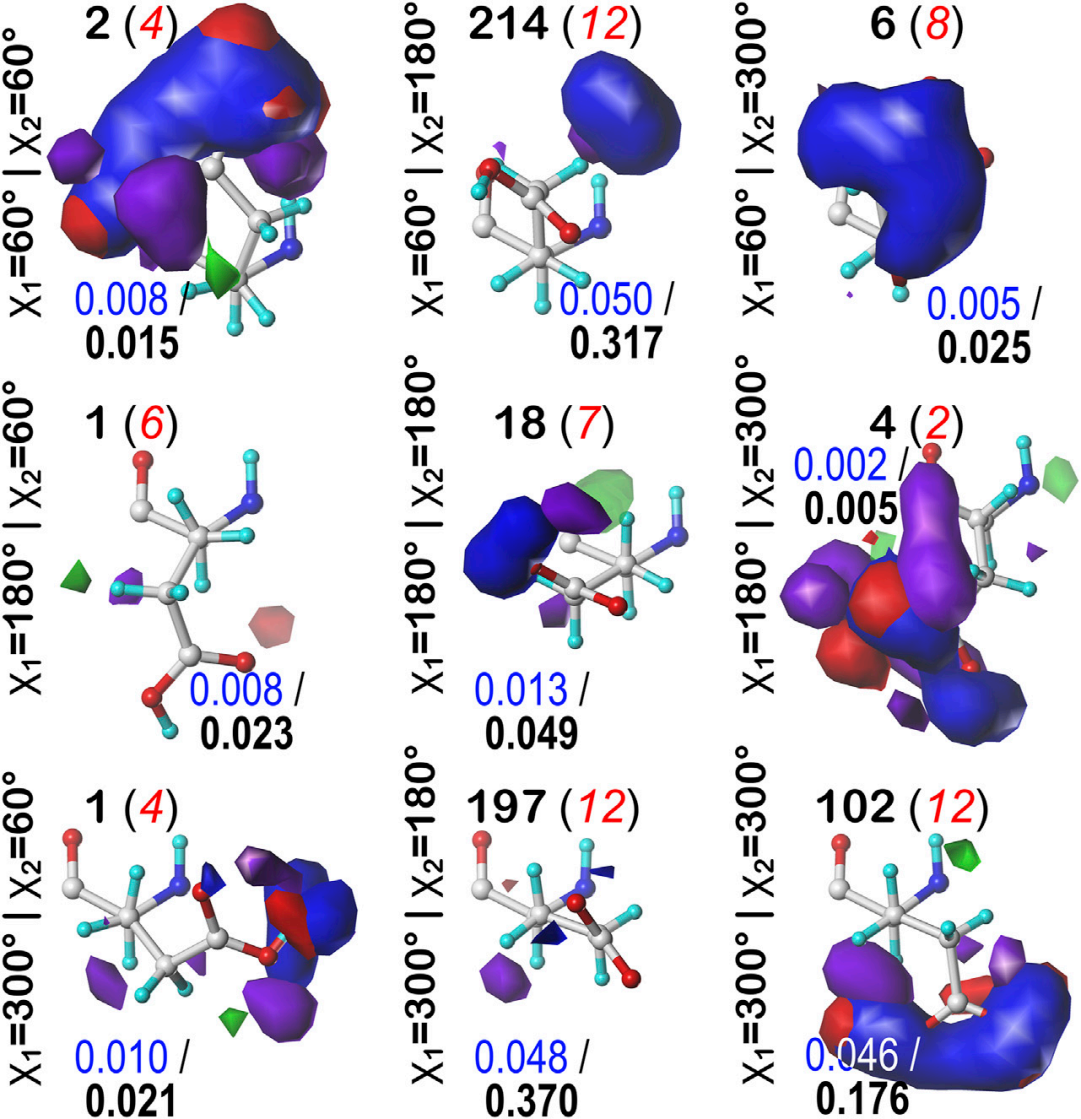
Hydropathic interaction maps displaying the Gaussian-weighted average sidechain environments of glutamic acid in the highest populated clusters of the nine parses of the *b1* chess square at pH = 4.224. Residues are oriented such that the CA-CB z-axis points upward and the x-axis runs to the right. The parses of the χ_1_ and χ_2_ angles are indicated along the side of each map. The cluster ID and number of clusters in the parse are given above the map in black and red, respectively. Below each map, in blue, is indicated the fraction of the entire chess square represented by each map, followed in black by the parse’s representative fraction of the chess square. Blue contours indicate position and magnitude of positive polar interactions near the sidechain, while red represents negative polar interactions. Green and purple contours indicate positive and negative hydrophobic interactions, respectively.

Glutamic acid tells a very similar story to that of aspartic acid, so many of the points made for that residue stand here, as well. First, the bulk of interactions made with the GLU sidechain are of the positive polar type, followed by negative polar. Again, many clusters were also calculated to have high SASA. Also, we calculated GLU maps with three times as many parses as ASP (*vide supra*), due to the 1-carbon extension to its sidechain, making the number of clusters about three times as many. We believed it is redundant to showcase maps for every average cluster in every subparse. Instead, we have chosen to focus on the ***b1*** chess square and show maps of its highest occupied clusters in each parse (Figure 10). This collection is representative of the 67 ***b1*** clusters, and suggests the diversity of sidechain orientations available in the full map set. One aspect of the GLU maps that we expected to see was an amplified presence of hydrophobic interactions compared to the ASP maps. However slightly, the maps of these specific clusters do show some indication of additional hydrophobic interactions localized around the hydrophobic chain, although these interactions appear more likely in the lower population parses. Their lack of visibility in Figure 10 may be more due to the limitations of contouring at consistent values than anything else, but perhaps the expected hydrophobic interactions with this sidechain are actually rare or have backbone conformation dependence. A confounding factor certainly is that GLU is even more solvent exposed than ASP, and this will be explored below. Numerical data for all GLU chess squares is provided in Supplementary Table S6.

#### Histidine

**FIGURE 11:**
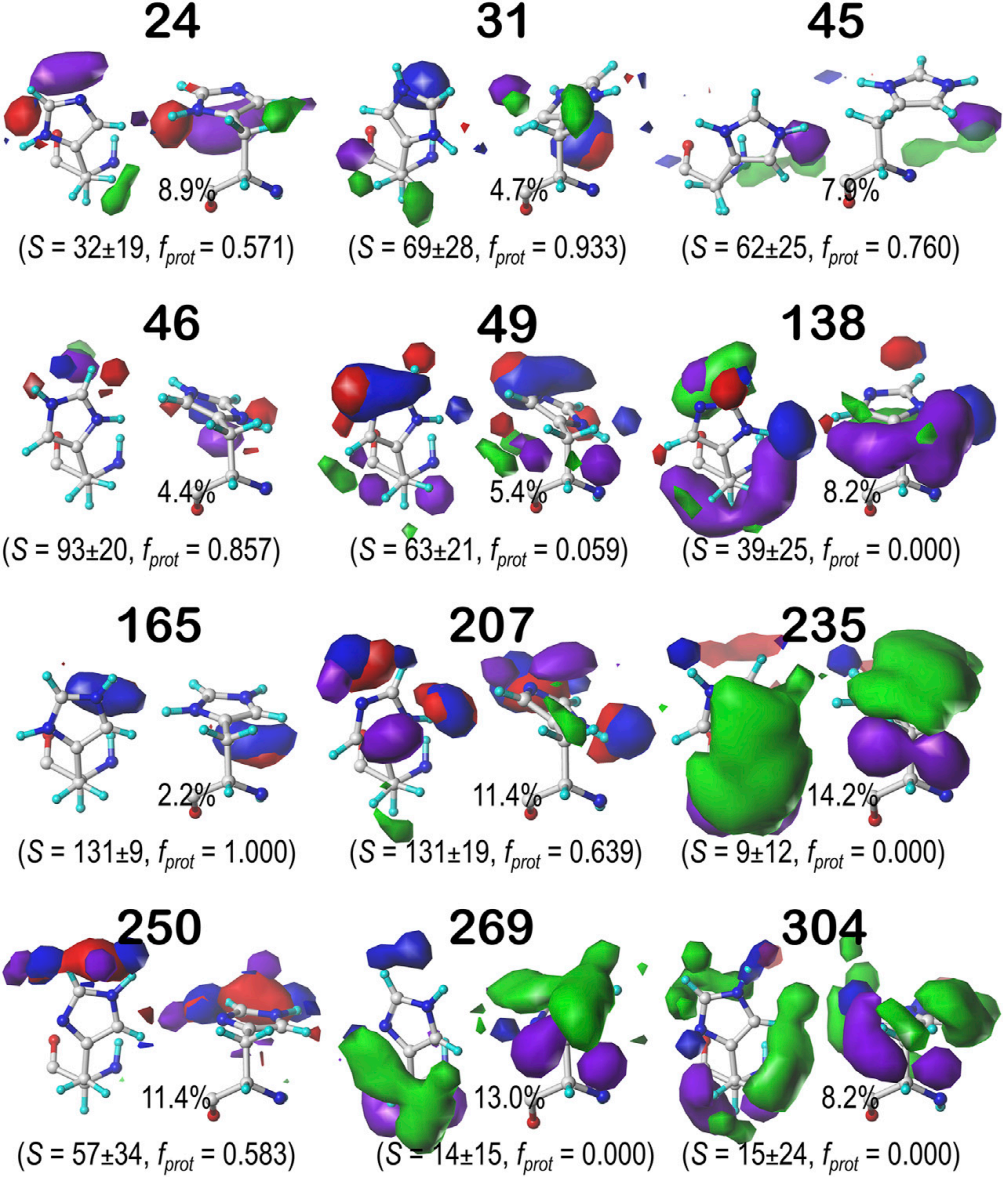
Hydropathic interaction maps displaying the Gaussian-weighted average sidechain environments of histidine in the χ_1_ = 60° parse of the *b1* chess square at pH = 5.174. See caption for Figure 7.

Histidine naturally tells very much a different story from ASP and GLU. Its imidazole sidechain can play numerous roles in protein structure. Not only does it have more protonation states than the acidic residues we have discussed, but its two nitrogens can act as either (or both) hydrogen bond donors and acceptors in any combination. Its ring is partially hydrophobic and aromatic, meaning it can make any variety of polar, nonpolar, and π-π stacking interactions with other residues. These π-π stacking interactions with aromatic residues, for example, may be indicated in maps where the ring is bordered by large, flat, green contours. This brand of versatility is very clearly indicated in our generated maps for HIS. Figure 11 displays the contour maps for the HIS ***b1.60*** chess square parse. Supplementary Figures S20–S30 for histidine maps in the ***b1.180***, ***b1.300*** parses and all parses of the ***c5***, ***d5*** and ***f6*** chess squares. The patterns in these maps are complex, but interpretable in terms of the interaction types. A detailed description for all 12 clustered maps in the 0.60 parse of the ***b1*** chess square would be too much for here, but first, it is clear that all maps displayed here (and in Supplementary Figures S20–S30) represent unique sets of interaction features, or routes to complete the residue's hydropathic valences. Consider cluster **31** in the ***b1.60*** map set (Figure 11): 93.3% of the histidines in this cluster are protonated, it has mid-range solvent exposure, the CB methylene is making hydrophobic interactions (green) with its environment, and the protonated NE is engaged in a hydrogen bonding interaction (blue) largely perpendicular to the ring. Cluster **235** here is singly protonated at NE, which enagages with an on-axis hydrogen bond, and has very low solvent exposure, and its environment is dominated by hydrophobic interactions, both favorable (green) and unfavorable (purple), with the former above the ring and the latter below the ring. Comprehensive numerical data for all chess squares of histidine is provided in Supplementary Table S7.

### Hydropathic Character of Maps With Changes in pH

**FIGURE 12:**
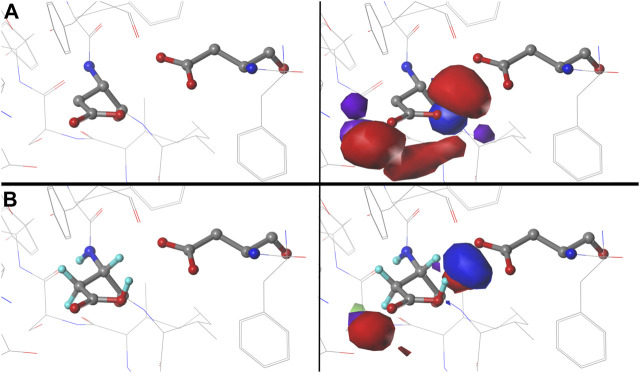
Variations in mapped environments around ASP141A in PDB structure 1WNS. **(A)** structure model mapped environment around deprotonated ASP141A with strong unfavorable polar interaction between it and nearby residue ASP215A (pH 9). **(B)** structure model and mapped environment around protonated ASP141A with new strong, favorable polar interaction with ASP215A (pH 5).

We were interested to see how changing the environmental pH would affect the maps. In other words, can we rationally “tune” the residue interactions by this means, and can that be exploited in protein design, e.g., to stabilize or destabilize binding sites, folds or interfaces? As an illustration, consider ASP141A in PDB structure 1WNS—family B DNA polymerase from hyperthermophilic archaeon pyrococcus kodakaraensis KOD1 (Hashimoto et al., 2001), which is situated in a highly anionic region with three other acidic residue side chains. This residue is in our cluster **202** of parse ***b1.180*** with *f*_*prot*_ = 0.520 and has a significant free energy difference between protonated and deprotonated states. Our model suggests ASP141A has an elevated pK_a_ and, when protonated, forms a hydrogen bond with ASP215A. There are significant visible differences between the calculated maps for this particular residue (Figure 12): at high pH (9), the interactions surrounding ASP141A (top) are largely unfavorable polar, but protonation, as shown in the low pH (5) case, protonates one of the carboxylate oxygens and yields a strong favorable hydrogen bond between it and ASP215A. As described earlier, the map contours displayed in this work were calculated at what we are calling pH_50_, which shows the highest diversity of protonated and deprotonated cases. Such maps can be calculated, clustered, etc. at any pH, and indeed making use of different maps at different protonation states will expand the scope for protein structure prediction of real situations where ionization states can vary due to local environments.

**FIGURE 13:**
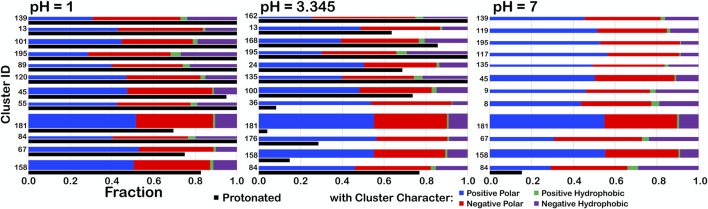
Character interaction charts for ASP residues in the b1.300 parse at pH 1, 3.345, and 7. The fraction of each interaction type is given on the x-axis, for each cluster ID on the y-axis. The bars are arranged such that, descending, clusters have smaller SASAs. The thickness of the bars indicates residue population contained within that cluster. The black bars indicate *f*_*prot*_, the fraction of the residues in the cluster protonated.

For further insight, we examined the interaction character of ASPs in one parse, ***b1*.300**, to determine if the relative fractions of our four-type quartet of interactions were altered with changes in pH (Figure 13). We expected to see small, but noticeable, changes in clustering of residues as adjustment of pH altered the memberships of the clusters as protonation became either more favorable or unfavorable. To facilitate comparisons between the cluster sets at different pH values, the bars are arranged by increasing average solvent-accessible surface area for the cluster (low to high). At pHs of 1, 3.345 (i.e., pH_50_) and 7, some character changes were in fact observed, but, interestingly, most of these occurred in low population clusters. We theorize that, as residues clustered differently, residues being added/subtracted to/from new groups simply had a greater impact on the overall character of smaller clusters. One point of note, however, is that, although most clusters with high SASA had the highest protonation levels (discussed later), only cluster **84** retained any level of protonation at pH 7, in spite of having the lowest SASA. This suggests that this cluster, in particular, describes scenarios where aspartate protonation is energetically required.

We also examined the interaction character of the GLU ***b1.300.180*** parse (Supplementary Figures S31), which is probably the parse most like the ***b1.300*** parse of ASP. The clusters within this GLU parse generally involved more hydrophobic interactions, both favorable and unfavorable, than those of the ASP ***b1.300*** parse. However, these observations are subtle and not easily visualized in the map contours. Nevertheless, overall, the average fractions of favorable and unfavorable hydrophobic interaction contributions, *f*_*hydro*(+)_ and *f*_*hydro*(–)_, are 0.038 and 0.218, respectively for GLU, and 0.021 and 0.153 for ASP at their respective pH_50_s. Importantly, the higher propensity for hydrophobic interactions by GLU, due to the additional methylene in the sidechain, are encoded in the interaction maps on a cluster by cluster basis.

Our ability to generate tunable maps for HIS is slightly more limited. The constrained conformational flexibility of the HIS sidechain and surrounding protein allowed by our approach could clearly be remedied by molecular dynamics or even energy minimization, but the cost–beyond CPU, etc. –would be the loss of positional certainty afforded by experimental data. That said, our map data for HIS, like ASP and GLU, exhaustively captures the many possible HIS interaction environments found in crystallographic structures exploitable for protein structure analyses and predictions.

### Solvent-Accessible Surface Areas for the Ionizable Residues

**FIGURE 14:**
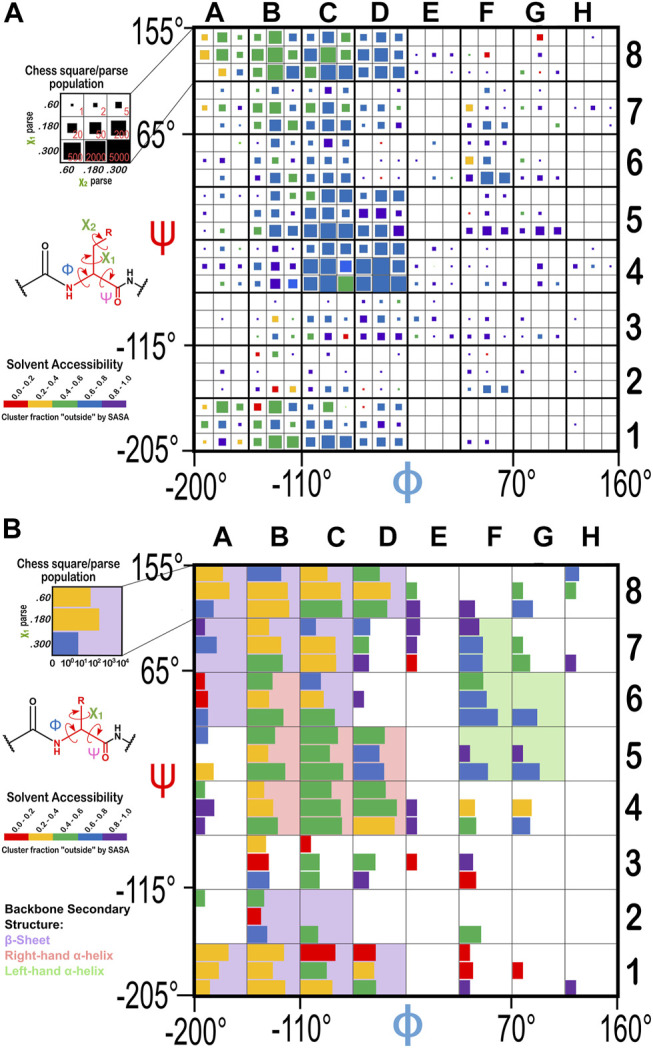
Ramachandran chessboard displaying the chess square/parse population for **(A)** glutamic acid and **(B)** histidine. The (χ_1_/χ_2_) parse populations for GLU are represented by colored squares with sizes as indicated on the legend. The (χ_1_) parse populations for HIS are represented in log_10_ scale with colored bars. See also caption for Figure 1.

The historical Ramachandran plots showed the relationship between backbone angles and frequency of observation. Our chessboard schema (Figure 1 for ASP, Figure 14 for GLU and HIS) was intended to organize our dataset by backbone structure, and thus facilitate comparisons between like residues. We also see a further population dependence on χ_1_ (and χ_2_ for GLU). In fact, further exploration revealed that solvent accessibility for each of our three residues is also seemingly dependent on the residue’s backbone and χ angles, which suggests a trend between this level of solvent exposure and underlying protein structure. For example, the average SASAs for ASP residues were calculated to be 37, 59, 64, and 64 Å^2^ for the ***b1***, ***c5***, ***d5***, and ***f6*** chess squares, respectively. With a similar trend, the average SASAs for GLU residues were calculated to be 57, 75, 80, and 81 Å^2^ for the ***b1***, ***c5***, ***d5***, and ***f6*** chess squares, respectively. However, in spite of it being significantly more hydrophobic than ASP and GLU, and thus more likely to be buried, GETAREA calculations for HIS yielded the surprisingly large average SASAs of 41, 59, 62, and 79 Å^2^ for the ***b1***, ***c5***, ***d5***, ***f6*** chess squares, respectively.

To evaluate our data in a more nuanced way, we calculated the “fraction outside” (*f*_*outside*_) metric based on GETAREA (Fraczkiewicz and Braun, 1998), as described in Methods. The *f*_*outside*_ values for each chess square/parse are also illustrated in Figures 1, 14, with the colors of the bars (that represent parse populations by their lengths) for ASP and HIS or squares (that represent parse populations by their areas) for GLU. Chess square/parses within the β-pleat region of the Ramachandran plot for aspartate (Figure 1), as expected, show lower *f*_*outside*_ (more buried) relative to the right- and left-hand α-helix, i.e., most parses show averaged *f*_*outside*_ in the 0.4–0.6 (green) range, whereas in the α-helix region most are in the *f*_*outside*_ range 0.6–0.8, and the left-hand α-helix is still more exposed, in the *f*_*outside*_ range 0.8–1. 0. The same trends hold for glutamates (Figure 14A), although the data suggests somewhat larger *f*_*outside*_ values. This is likely a result of GLU’s inherent additional surface area concomitant with its 1-carbon chain extension. The *f*_*outside*_ trends for HIS (Figure 14B) suggest more buriedness: in the β-pleat region of the Ramachandran plot, the parses are evenly split between the 0.2–0.4 and 0.4–0.6 ranges (yellow and green), histidines in the α-helix region are in the f_outside_ range 0.4–0.6, while those in the left-hand α-helix are more exposed, in the range 0.6–0.8.

It should be noted that the sidechain solvent-accessible surface areas for these three residues in Gly-X-Gly “random coil” tripeptides show that histidine has a larger surface area (154.6 Å^2^) than either aspartate (113.0 Å^2^) or glutamate (141.2 Å^2^) (Fraczkiewicz and Braun, 1998), which is incorporated into the *f*_*outside*_ calculations. Thus, while HIS may have, overall, higher solvent exposure in surface area, the actual fraction of solvent-exposed residues is smaller. All three residues show the same trend: larger solvent exposure in the α-helix regions that is more extreme in the left-hand region, and greater burial in the β-pleat region. These conclusions are in qualitative agreement with those of Lins et al. (2003) in their report on differences in solvent-accessible surface area between residues in different secondary structures. However, *f*_*outside*_, exactly as SASA does, varies from cluster-to-cluster within each chess square and parse. For example, *f*_*outside*_ for ASP ***b1.300*** ranges widely–between 0.077 (cluster **84**) to 1.000 (cluster **162**), despite its overall *f*_*outside*_ of <0.4 suggesting mostly burial for this group of residues.

The SASA and *f*_*outside*_ values for all three residues in this study, on a cluster-by-cluster basis are included in the Supplementary Tables S5–S7. To summarize, each 3D map cluster represents a unique set of interactions that also encodes solvent exposure and buriedness. We should emphasize that map profiles *appearing* to be similar could manifest with different buriedness and/or protonation, and thus remain unique.

### Summary and Conclusion

We analyzed the interaction environments of more than 105,000 ionizable amino acid residues (aspartic acid, glutamic acid, histidine) in a diverse collection of protein structures. From above and our previous reports (Ahmed et al., 2015; Ahmed et al., 2019), it is clear that the hydropathic environment surrounding an amino acid residue in a protein can be mapped in terms of its interactions. Significantly, the patterns of interactions within the maps, representing the constellation of contacts and their interaction strengths and characters, cluster into a fairly limited set of unique, backbone-dependent motifs. Each of these motifs can be rendered into an average map quartet and an average prototype residue structure. Thus, we have produced a backbone-dependent library of not only sidechain rotamers, but also 3D residue interaction preferences. The presence of a feature, such as a favorable polar interaction in one of these maps, e.g., an ASP in the ***b1.300*** (β-pleat) cluster **100** (Figure 9), where the carboxylate/carboxylic acid functional group is involved in hydrogen bonding through both oxygens, should have complementary donors/acceptors on neighboring residue(s). Accordingly, those residue's maps should contain similar features, and the alignment of these features–and all others from a collection of such maps–would describe a well-organized hydropathic interaction network.

It is not just the favorable hydrophobic and polar interactions that constitute this network. The maps illustrated by contours here, and previously (Ahmed et al., 2015; Ahmed et al., 2019; AL Mughram et al., 2021), nearly ubiquitously display unfavorable polar and hydrophobic interactions. These interactions are integral parts of protein structure; for example, even polar residues like the ASP, GLU, and HIS of this report have hydrophobic atoms covalently bonded to the polar functional groups. Thus, a background of unfavorable hydrophobic interactions is usually seen with strong favorable polar interactions. However, other hydrophobic interactions are functional components of structure that Nature uses, e.g., for adding flexibility or isolating water. Developing an understanding of them will help illuminate protein design and drug discovery. Unfavorable polar interactions, on the other hand, provide a route to understanding and predicting residue ionization states. The presence of this type of interaction signals an opportunity for water intervention, an adjustment in local pH or can be used as drug design cues.

While our predictions of pK_a_s for ASP and GLU are adequate (and seemingly less so for HIS over a much smaller training set), our primary goal was not that, but instead to evaluate the hydropathic environments surrounding these residue types. As expected, those environments change drastically with pH. We illustrated environments with 3D maps for an artificial half-way point–pH_50_–that showed a range of environments, but we have also calculated maps for other pH cases, and the nature of interactions displayed therein are, although unsurprising, quite informative. Importantly, this means that we can *tune* residue hydropathic environment maps as a function of pH, and that they encode this critical element of structure, interaction and energetics in a rational way. Thus, if we use these maps as part of a scheme for protein structure building and prediction, we have the additional scope to explore ionization states in understanding and defining optimal protein structures.

In our 2019 report (Ahmed et al.), we stated that full understanding of the individual environment maps for alanine would first require completing the analysis for all residue types. This current report is a status update on that task–for ASP, GLU and HIS. The remaining residues are in various stages of completion and analysis, and we anticipate additional communications in the near future.

As with alanine, our evaluation of interactions of the ionizable residues with 3D maps backs our interaction homology paradigm–for understanding and potentially predicting protein structure. The hydropathic valence for ASP and GLU is largely satisfied by a functional group that complements the carboxy acid, and some involvement with the CB, CG (and for GLU, the CD) methylenes by a hydrophobic interaction partner, except if the sidechain is fully solvent exposed. HIS is, however, much more complex, involving additional terms such as hydrophobic interactions with aromatic carbons that may be of π-π character and polar interactions that include hydrogen bonding with its ND1 and/or NE2, as either acceptors or donors. As these effects are recorded within the maps, we see that it is the hydropathic “field” of the atoms surrounding a residue, not specific residue types or atoms, that directs its conformation or other properties, including rotameric and secondary structure. Finally, biological structure is a puzzle consisting of a delicate balance of effects, mostly favorable but others seemingly counterproductive. Assembing structure by homology modeling (Eisenmenger et al., 1993; Laughton, 1994; Krivov et al., 2009) or even *de novo* structure prediction (Alley et al., 2019; Senior et al., 2020; Yang et al., 2020) involves many puzzle pieces and interactions, but some key information involving, e.g., hydrophobic interactions or residue ionizations is not utilized in the usual Newtonian physics-based approaches.

Our ability to map interactions in 3D space, including a rational means to explore the local pH of individual residues in more or less real time should be advantageous in later studies. Since the maps highlight *interactions*, building structural models that optimize the map-map overlaps of interactions arising from adjacent or through-space residue map pairs (or larger sets) could yield a very useful and unique target function for protein structure prediction, likely quite amenable for machine learning optimization.

## Data Availability

The datasets presented in this study can be found in online repositories. The names of the repository/repositories and accession number(s) can be found in the article/Supplementary Material.
