# Supplementary material for: 3D Interaction Homology: Computational Titration of Aspartic Acid, Glutamic Acid and Histidine Can Create pH-Tunable Hydropathic Environment Maps
Source: Front Mol Biosci. 2021 Nov 3;8:773385. doi: 10.3389/fmolb.2021.773385 (PMC8595396; doi:10.3389/fmolb.2021.773385)
Supplement: Supplementary file 2 [file Table4.pdf]

**Table S4: Number of clusters in each chess square and parse for ASP, GLU and HIS.**

| | | | | | | | | |
|---|---|---|---|---|---|---|---|---|
| | **Number of aspartate clusters in parses** | | | **60 / 180 / 300** | | | | |
| | ***a*** | ***b*** | ***c*** | ***d*** | ***e*** | ***f*** | ***g*** | ***h*** |
| ***1*** | 12/12/5 | 12/12/12 | 12/12/12 | 9/7/10 | 0/0/0 | 1/1/3 | 0/2/1 | 0/0/1 |
| ***2*** | 4/2/0 | 8/2/2 | 6/4/3 | 0/1/1 | 0/1/1 | 1/5/11 | 0/0/0 | 0/0/0 |
| ***3*** | 0/1/0 | 1/5/3 | 3/5/6 | 3/5/5 | 1/1/1 | 1/2/6 | 0/1/1 | 0/1/0 |
| ***4*** | 1/3/1 | 3/8/11 | 12/12/12 | 12/12/12 | 1/1/2 | 0/1/4 | 0/3/3 | 0/1/1 |
| ***5*** | 3/3/1 | 12/7/12 | 12/12/12 | 11/10/12 | 0/0/0 | 2/6/12 | 3/5/12 | 2/1/1 |
| ***6*** | 3/5/2 | 12/12/8 | 12/11/12 | 1/2/2 | 1/1/1 | 3/12/12 | 1/5/5 | 1/0/0 |
| ***7*** | 1/11/2 | 5/12/12 | 7/12/12 | 2/9/5 | 1/1/1 | 2/10/9 | 0/4/1 | 0/1/1 |
| ***8*** | 5/12/4 | 11/12/12 | 12/12/12 | 7/12/12 | 1/1/1 | 0/1/1 | 0/1/1 | 0/1/1 |
| | **Number of glutamate clusters in parses** | | | **60.60 / 60.180 / 60.300<br>180.60 / 180.180 / 180.300<br>300.60 / 300.180 / 300.300** | | | | |
| | ***a*** | ***b*** | ***c*** | ***d*** | ***e*** | ***f*** | ***g*** | ***h*** |
| ***1*** | 1/12/4<br>4/4/1<br>1/5/1 | 4/12/8<br>6/7/2<br>4/12/12 | 3/12/6<br>5/6/1<br>11/12/12 | 1/4/3<br>4/5/1<br>4/9/5 | 0/0/0<br>0/0/0<br>0/0/0 | 0/0/0<br>0/0/0<br>1/1/0 | 0/0/0<br>0/0/0<br>0/0/0 | 0/0/0<br>1/0/0<br>0/0/0 |
| ***2*** | 0/0/0<br>0/1/0<br>0/1/0 | 1/3/1<br>1/0/0<br>1/3/2 | 0/1/0<br>1/0/1<br>1/3/2 | 0/0/1<br>0/1/0<br>1/1/1 | 0/0/0<br>0/0/0<br>0/0/0 | 1/1/0<br>1/1/0<br>1/6/5 | 0/0/0<br>1/0/0<br>0/0/0 | 0/0/0<br>1/0/0<br>0/0/0 |
| ***3*** | 0/0/0<br>0/1/0<br>0/0/0 | 0/1/0<br>1/1/1<br>1/1/1 | 0/1/0<br>4/4/1<br>3/5/1 | 1/1/1<br>5/6/1<br>3/5/4 | 0/1/0<br>1/1/0<br>1/1/1 | 0/0/0<br>1/1/0<br>1/1/1 | 0/0/0<br>1/0/0<br>1/1/1 | 0/0/0<br>1/0/0<br>0/0/0 |
| ***4*** | 1/1/1<br>2/3/1<br>0/1/1 | 1/3/1<br>7/7/3<br>2/9/9 | 5/12/12<br>12/12/12<br>12/12/12 | 6/12/12<br>12/12/12<br>12/12/12 | 0/1/1<br>1/1/1<br>1/1/1 | 0/1/0<br>1/1/0<br>0/1/1 | 0/0/0<br>0/3/0<br>0/1/1 | 0/1/0<br>1/1/1<br>0/0/0 |
| ***5*** | 1/1/1<br>1/0/0<br>1/1/0 | 1/7/3<br>4/6/1<br>4/12/10 | 9/12/12<br>12/12/6<br>12/12/12 | 3/12/12<br>9/12/3<br>9/12/12 | 0/0/0<br>0/0/0<br>0/0/0 | 0/1/1<br>1/1/0<br>3/9/4 | 0/0/0<br>1/1/0<br>2/6/5 | 0/0/0<br>0/0/0<br>0/0/0 |
| ***6*** | 0/0/1<br>1/2/0<br>0/1/0 | 1/6/1<br>3/3/1<br>2/10/5 | 1/6/4<br>5/4/3<br>4/10/11 | 0/1/0<br>0/1/1<br>1/1/1 | 0/1/0<br>1/0/0<br>0/1/1 | 1/2/1<br>4/4/0<br>4/12/10 | 0/0/0<br>1/1/0<br>1/2/1 | 0/0/0<br>0/0/0<br>0/0/0 |
| ***7*** | 0/1/0<br>1/4/1<br>0/1/1 | 1/4/1<br>9/9/4<br>4/12/6 | 1/4/3<br>10/12/6<br>9/12/12 | 0/0/1<br>4/5/1<br>1/4/3 | 0/0/0<br>0/0/0<br>0/0/0 | 0/2/1<br>3/1/1<br>1/6/3 | 0/1/0<br>1/1/0<br>0/0/1 | 0/0/0<br>1/1/1<br>0/0/1 |
| ***8*** | 3/9/1<br>10/12/3<br>0/4/3 | 4/12/5<br>12/12/5<br>8/12/12 | 5/12/8<br>12/12/12<br>11/12/12 | 4/12/6<br>12/12/6<br>9/12/12 | 0/0/0<br>1/1/1<br>1/0/1 | 0/0/0<br>1/1/0<br>1/1/0 | 0/1/0<br>0/1/0<br>1/1/1 | 0/1/0<br>0/0/0<br>0/0/0 |
| | **Number of histidine clusters in parses** | | | **60 / 180 / 300** | | | | |
| | ***a*** | ***b*** | ***c*** | ***d*** | ***e*** | ***f*** | ***g*** | ***h*** |
| ***1*** | 9/5/1 | 12/6/12 | 9/7/10 | 3/2/5 | 0/0/0 | 1/1/1 | 0/1/0 | 0/0/1 |
| ***2*** | 1/0/0 | 1/1/3 | 0/0/2 | 0/0/0 | 0/0/0 | 0/0/3 | 0/0/0 | 0/0/0 |
| ***3*** | 0/0/0 | 1/3/3 | 1/2/2 | 0/4/1 | 0/1/0 | 0/1/1 | 0/0/0 | 0/0/0 |
| ***4*** | 1/2/1 | 1/7/9 | 10/12/12 | 11/12/12 | 0/1/1 | 0/1/1 | 0/2/2 | 0/0/0 |
| ***5*** | 1/0/2 | 7/3/12 | 12/8/12 | 10/6/8 | 0/0/0 | 0/1/9 | 0/1/7 | 0/0/0 |
| ***6*** | 1/1/1 | 5/3/12 | 3/4/9 | 0/1/0 | 0/0/0 | 5/8/12 | 0/0/7 | 0/0/0 |
| ***7*** | 1/4/0 | 4/12/12 | 1/12/11 | 1/1/1 | 1/1/1 | 2/4/5 | 0/1/2 | 0/0/1 |
| ***8*** | 8/12/3 | 12/12/12 | 6/12/12 | 6/12/10 | 0/1/1 | 0/0/1 | 0/1/3 | 1/1/0 |
