# Supplementary material for: 3D Interaction Homology: Computational Titration of Aspartic Acid, Glutamic Acid and Histidine Can Create pH-Tunable Hydropathic Environment Maps
Source: Front Mol Biosci. 2021 Nov 3;8:773385. doi: 10.3389/fmolb.2021.773385 (PMC8595396; doi:10.3389/fmolb.2021.773385)
Supplement: Supplementary file 3 [file Table2.pdf]

Table S2: Number of residues in each chess square and parse for ASP, GLU and HIS.

	**Number of aspartates in parses**			**60 / 180 / 300**				
	a	***b***	***c***	***d***	***e***	***f***	***g***	***h***
1	261/342/22	556/323/200	1162/260/650	39/37/120	0/0/0	3/2/15	0/5/4	0/0/1
2	14/8/0	47/6/7	33/16/13	0/2/1	0/1/1	4/29/56	0/0/0	0/0/0
3	0/3/0	4/25/8	10/20/34	11/15/19	2/1/2	3/8/25	0/2/3	0/1/0
4	5/9/2	10/44/76	279/833/4142	395/1578/6215	4/1/7	0/2/14	0/12/10	0/2/8
5	10/11/3	647/60/263	2539/309/3409	169/92/477	0/0/0	6/22/160	8/21/111	5/2/1
6	8/18/5	326/181/131	313/142/274	2/5/7	2/1/3	10/492/1035	1/21/25	1/0/0
7	2/163/6	23/1279/126	43/2250/254	6/67/23	3/5/6	7/65/74	0/18/4	0/2/1
8	27/292/13	118/1035/615	168/2483/2284	44/854/919	1/2/3	0/4/3	0/2/4	0/2/1
	**Number of glutamates in parses**			**60.60 / 60.180 / 60.300<br>180.60 / 180.180 / 180.300<br>300.60 / 300.180 / 300.300**				
	a	***b***	***c***	***d***	***e***	***f***	***g***	***h***
1	4/218/15<br>16/24/2<br>2/22/4	20/420/33<br>30/65/6<br>28/490/233	7/95/35<br>35/40/2<br>66/538/279	1/18/12<br>13/16/3<br>22/50/24	0/0/0<br>0/0/0<br>0/0/0	0/0/0<br>0/0/0<br>3/3/0	0/0/0<br>0/0/0<br>0/0/0	0/0/0<br>1/0/0<br>0/0/0
2	0/0/0<br>0/3/0<br>0/1/0	3/8/1<br>1/0/0<br>3/10/6	0/2/0<br>3/0/1<br>4/9/6	0/0/1<br>0/1/0<br>1/1/1	0/0/0<br>0/0/0<br>0/0/0	1/1/0<br>3/1/0<br>2/25/17	0/0/0<br>2/0/0<br>0/0/0	0/0/0<br>1/0/0<br>0/0/0
3	0/0/0<br>0/1/0<br>0/0/0	0/1/0<br>1/10/1<br>1/8/2	0/1/0<br>12/14/2<br>10/18/5	2/2/2<br>18/19/2<br>9/23/14	0/1/0<br>5/4/0<br>2/1/3	0/0/0<br>1/3/0<br>3/7/1	0/0/0<br>2/0/0<br>1/4/2	0/0/0<br>1/0/0<br>0/0/0
4	1/4/1<br>5/8/3<br>0/3/2	1/10/1<br>22/43/9<br>7/95/58	25/299/133<br>1135/1983/224<br>698/3463/1799	32/374/212<br>1393/4345/325<br>1139/4563/186<br>8	0/3/1<br>2/2/1<br>2/2/1	0/1/0<br>1/1/0<br>0/3/3	0/0/0<br>0/8/0<br>0/5/2	0/1/0<br>4/2/1<br>0/0/0
5	1/4/1<br>2/0/0<br>1/4/0	1/62/10<br>14/30/3<br>23/261/87	41/538/438<br>174/252/25<br>465/2053/1089	11/219/153<br>54/122/9<br>152/311/122	0/0/0<br>0/0/0<br>0/0/0	0/3/2<br>1/4/0<br>11/56/19	0/0/0<br>2/2/0<br>6/46/21	0/0/0<br>0/0/0<br>0/0/0
6	0/0/1<br>2/6/0<br>0/4/0	1/41/3<br>11/12/3<br>6/111/34	5/42/15<br>20/19/8<br>30/119/68	0/1/1<br>0/1/1<br>1/1/1	0/2/0<br>1/0/0<br>0/2/1	2/5/3<br>27/23/0<br>17/378/145	0/0/0<br>3/1/0<br>2/7/2	0/0/0<br>0/0/0<br>0/0/0
7	0/2/0<br>6/15/3<br>0/3/1	2/17/1<br>64/201/17<br>18/223/78	2/15/7<br>69/208/28<br>38/262/109	0/0/2<br>11/16/3<br>2/16/8	0/4/1<br>1/0/0<br>2/3/0	0/6/1<br>15/2/1<br>4/33/11	0/1/0<br>2/3/0<br>0/0/3	0/0/0<br>1/4/1<br>0/0/1
8	10/84/4<br>61/234/10<br>0/23/10	15/442/25<br>251/1511/55<br>56/1852/419	19/213/56<br>404/1854/84<br>181/1733/779	14/91/23<br>193/645/43<br>87/376/217	0/0/0<br>1/3/2<br>2/0/1	0/0/0<br>1/6/0<br>1/1/0	0/1/0<br>0/3/0<br>3/1/2	0/1/0<br>0/0/0<br>0/0/0
	**Number of histidines in parses**			**60 / 180 / 300**				
	a	***b***	***c***	***d***	***e***	***f***	***g***	***h***
1	192/22/3	316/27/416	119/35/226	14/10/15	0/0/0	1/2/1	0/1/0	0/0/1
2	1/0/0	4/2/8	0/0/5	0/0/0	0/0/0	0/0/12	0/0/0	0/0/0
3	0/0/0	6/11/13	1/7/7	0/13/3	0/1/0	0/2/4	0/0/0	0/0/0
4	1/5/1	4/30/80	94/739/947	125/1504/905	0/1/1	0/3/4	0/7/5	0/0/0
5	2/0/7	45/10/431	395/69/1249	98/32/86	0/0/0	0/1/55	0/1/42	0/0/0
6	1/2/2	25/12/282	9/17/206	0/1/0	0/0/0	20/43/503	0/0/24	0/0/0
7	1/14/0	12/176/251	3/231/259	4/3/3	2/1/1	5/18/17	0/1/5	0/0/1
8	54/229/7	172/759/1009	39/748/1025	34/375/171	0/1/2	0/0/3	0/1/9	2/1/0
