# Supplementary material for: 3D Interaction Homology: Computational Titration of Aspartic Acid, Glutamic Acid and Histidine Can Create pH-Tunable Hydropathic Environment Maps
Source: Front Mol Biosci. 2021 Nov 3;8:773385. doi: 10.3389/fmolb.2021.773385 (PMC8595396; doi:10.3389/fmolb.2021.773385)
Supplement: Supplementary file 7 [file DataSheet1.pdf]

3D Interaction Homology: Computational Titration of Aspartic Acid, Glutamic Acid and Histidine Residues Can Create pH-Tunable Hydropathic Environment Maps

Noah B. Herrington[1] and Glen E. Kellogg[1,2,*]

From the [1]*Department of Medicinal Chemistry and Institute for Structural Biology, Drug Discovery and Development, Virginia Commonwealth University, Richmond, Virginia, USA;* [2]*Center for the Study of Biological Complexity, Virginia Commonwealth University, Richmond, Virginia, USA*

This Document:

Figure S1. Hydropathic interaction maps displaying the Gaussian-weighted average sidechain environments of aspartic acid in the χ_1 = 60° parse of the ***c5*** chess square at pH = 3.345.

Figure S2. Hydropathic interaction maps displaying the Gaussian-weighted average sidechain environments of aspartic acid in the χ_1 = 180° parse of the ***c5*** chess square at pH = 3.345.

Figure S3. Hydropathic interaction maps displaying the Gaussian-weighted average sidechain environments of aspartic acid in the χ_1 = 300° parse of the ***c5*** chess square at pH = 3.345.

Figure S4. Hydropathic interaction maps displaying the Gaussian-weighted average sidechain environments of aspartic acid in the χ_1 = 60° parse of the ***d5*** chess square at pH = 3.345.

Figure S5. Hydropathic interaction maps displaying the Gaussian-weighted average sidechain environments of aspartic acid in the χ_1 = 180° parse of the ***d5*** chess square at pH = 3.345.

Figure S6. Hydropathic interaction maps displaying the Gaussian-weighted average sidechain environments of aspartic acid in the χ_1 = 300° parse of the ***d5*** chess square at pH = 3.345.

Figure S7. Hydropathic interaction maps displaying the Gaussian-weighted average sidechain environments of aspartic acid in the χ_1 = 60° parse of the ***f6*** chess square at pH = 3.345.

Figure S8. Hydropathic interaction maps displaying the Gaussian-weighted average sidechain environments of aspartic acid in the χ_1 = 180° parse of the ***f6*** chess square at pH = 3.345.

Figure S9. Hydropathic interaction maps displaying the Gaussian-weighted average sidechain environments of aspartic acid in the χ_1 = 300° parse of the ***f6*** chess square at pH = 3.345.

Figure S10. Hydropathic interaction maps displaying the Gaussian-weighted average sidechain environments of histidine in the χ_1 = 180° parse of the ***b1*** chess square at pH = 3.345.

Figure S11. Hydropathic interaction maps displaying the Gaussian-weighted average sidechain environments of histidine in the χ_1 = 300° parse of the ***b1*** chess square at pH = 3.345.

Figure S12. Hydropathic interaction maps displaying the Gaussian-weighted average sidechain environments of histidine in the χ_1 = 60° parse of the ***c5*** chess square at pH = 3.345.

Figure S13. Hydropathic interaction maps displaying the Gaussian-weighted average sidechain environments of histidine in the χ_1 = 180° parse of the ***c5*** chess square at pH = 3.345.

Figure S14. Hydropathic interaction maps displaying the Gaussian-weighted average sidechain environments of histidine in the χ_1 = 300° parse of the ***c5*** chess square at pH = 3.345.

Figure S15. Hydropathic interaction maps displaying the Gaussian-weighted average sidechain environments of histidine in the χ_1 = 60° parse of the ***d5*** chess square at pH = 3.345.

Figure S16. Hydropathic interaction maps displaying the Gaussian-weighted average sidechain environments of histidine in the χ_1 = 180° parse of the ***d5*** chess square at pH = 3.345.

Figure S17. Hydropathic interaction maps displaying the Gaussian-weighted average sidechain environments of histidine in the χ_1 = 300° parse of the ***d5*** chess square at pH = 3.345.

Figure S18. Hydropathic interaction maps displaying the Gaussian-weighted average sidechain environments of histidine in the χ_1 = 60° parse of the ***f6*** chess square at pH = 3.345.

Figure S19. Hydropathic interaction maps displaying the Gaussian-weighted average sidechain environments of histidine in the χ_1 = 180° parse of the ***f6*** chess square at pH = 3.345.

Figure S20. Hydropathic interaction maps displaying the Gaussian-weighted average sidechain environments of histidine in the χ_1 = 300° parse of the ***f6*** chess square at pH = 3.345.

Figure S21: Character interaction chart for the GLU ***b1.300.180*** parse at pH 4.224.

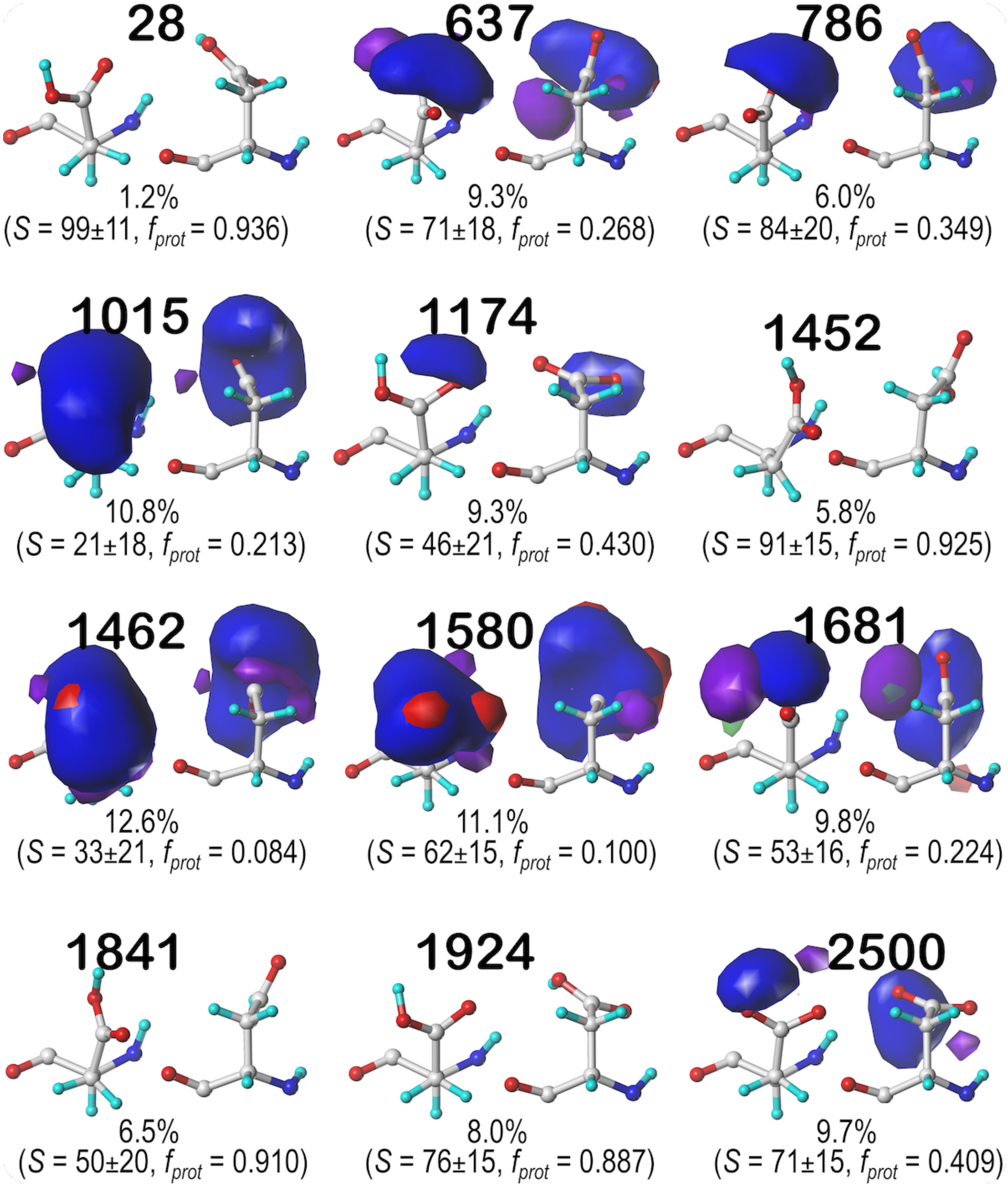

Figure S1: Hydropathic interaction maps displaying the Gaussian-weighted average sidechain environments of aspartic acid in the χ_1 = 60° parse of the *c5* chess square at pH = 3.345. Two map viewpoints are given for each cluster, whose ID is given in bold. The left map in each pair is oriented such that the CA-CB z-axis bond points upward, while the right is oriented to point it out of the page. The x-axis is oriented horizontally in both. The percentage indicates the fraction of the parse represented by that cluster. *S* represents the solvent accessible surface area in Å^2, and f_{prot} indicates the fraction of the cluster protonated at pH_{50}. Blue contours indicate positive polar interactions made with the sidechain, and red indicates negative polar interactions, while green and purple indicate positive and negative hydrophobic interactions, respectively.

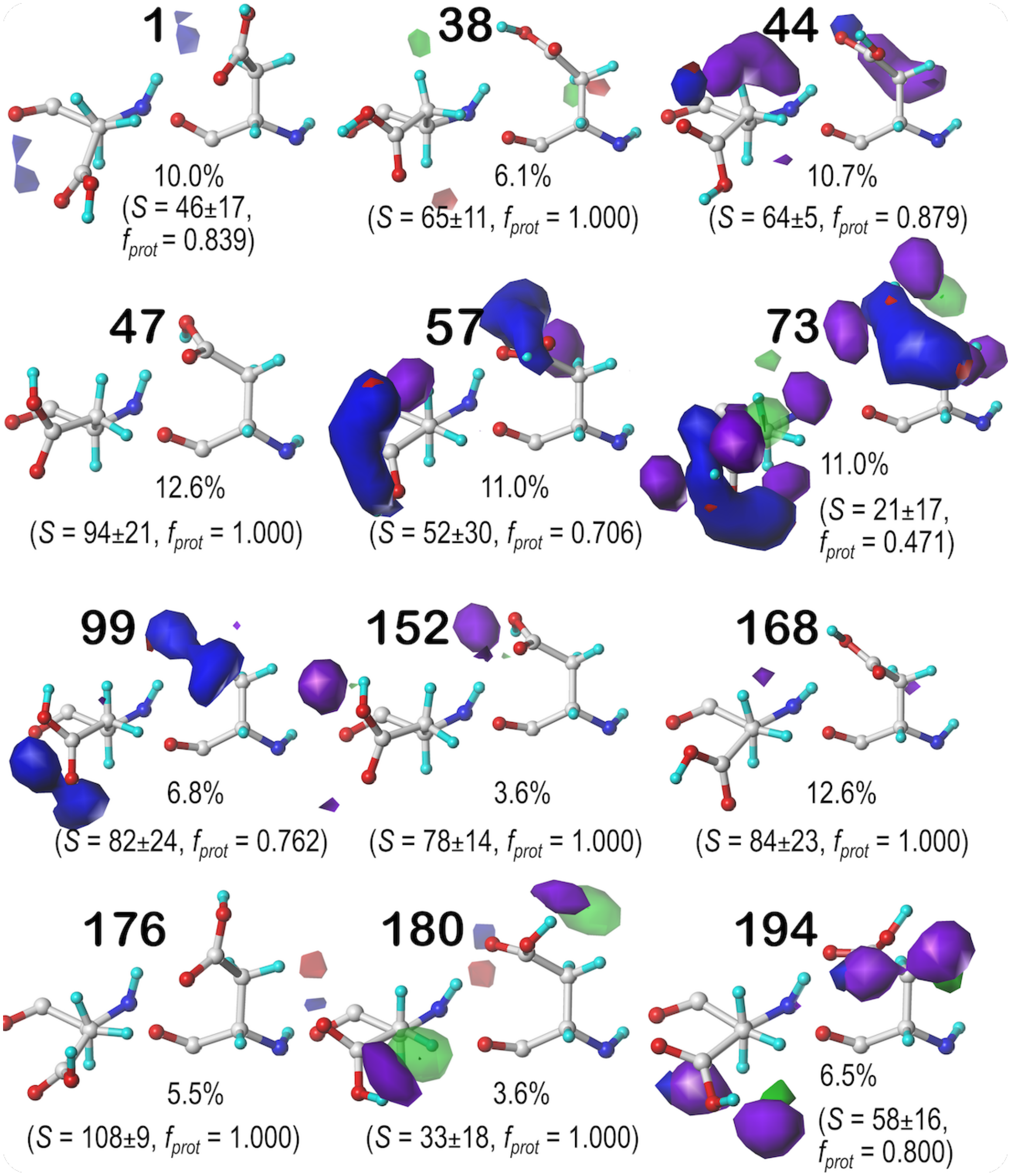

Figure S2: Hydropathic interaction maps displaying the Gaussian-weighted average sidechain environments of aspartic acid in the χ_1 = 180° parse of the *c5* chess square at pH = 3.345. See caption for Figure S1.

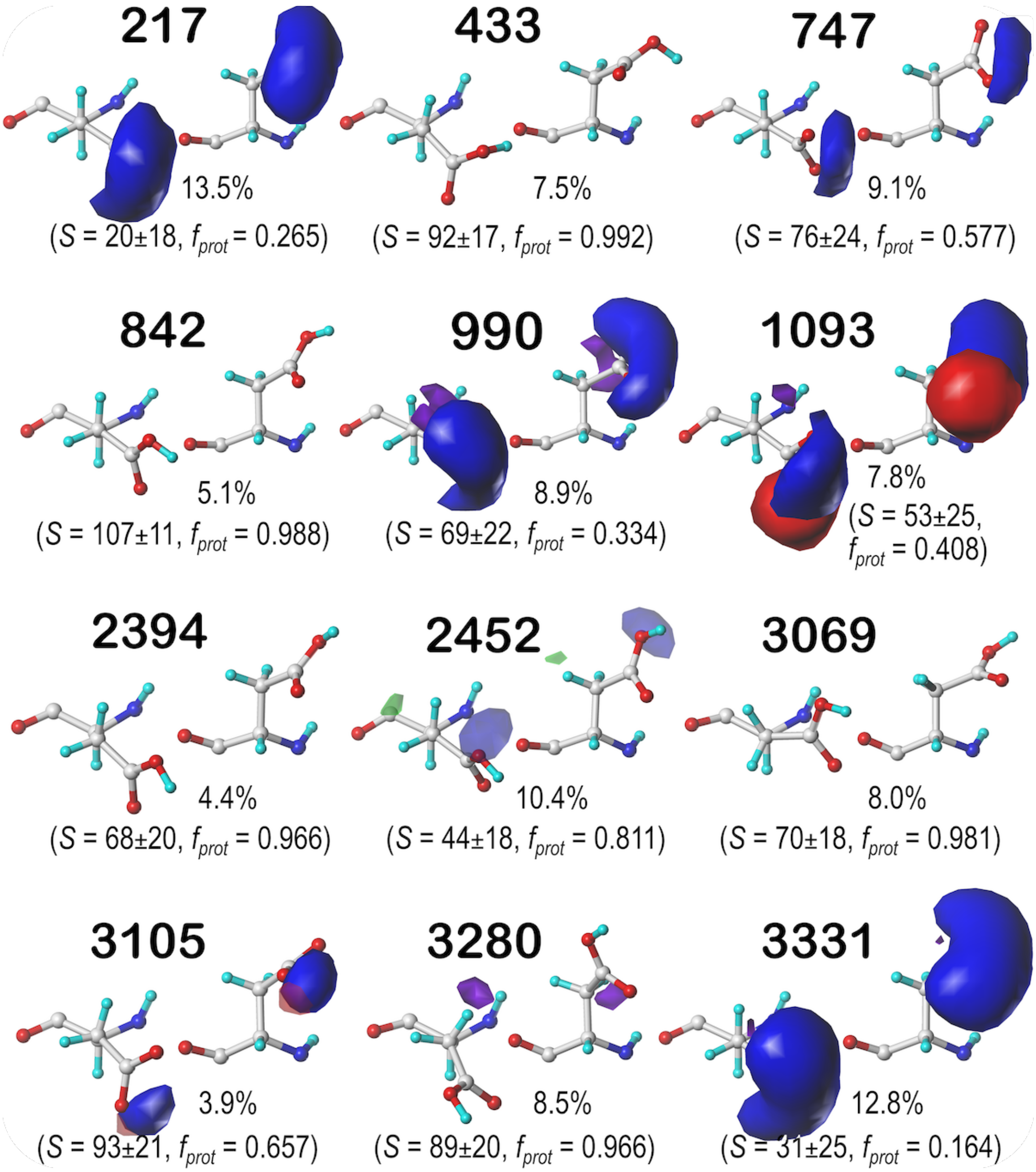

Figure S3: Hydropathic interaction maps displaying the Gaussian-weighted average sidechain environments of aspartic acid in the χ_1 = 300° parse of the *c5* chess square at pH = 3.345. See caption for Figure S1.

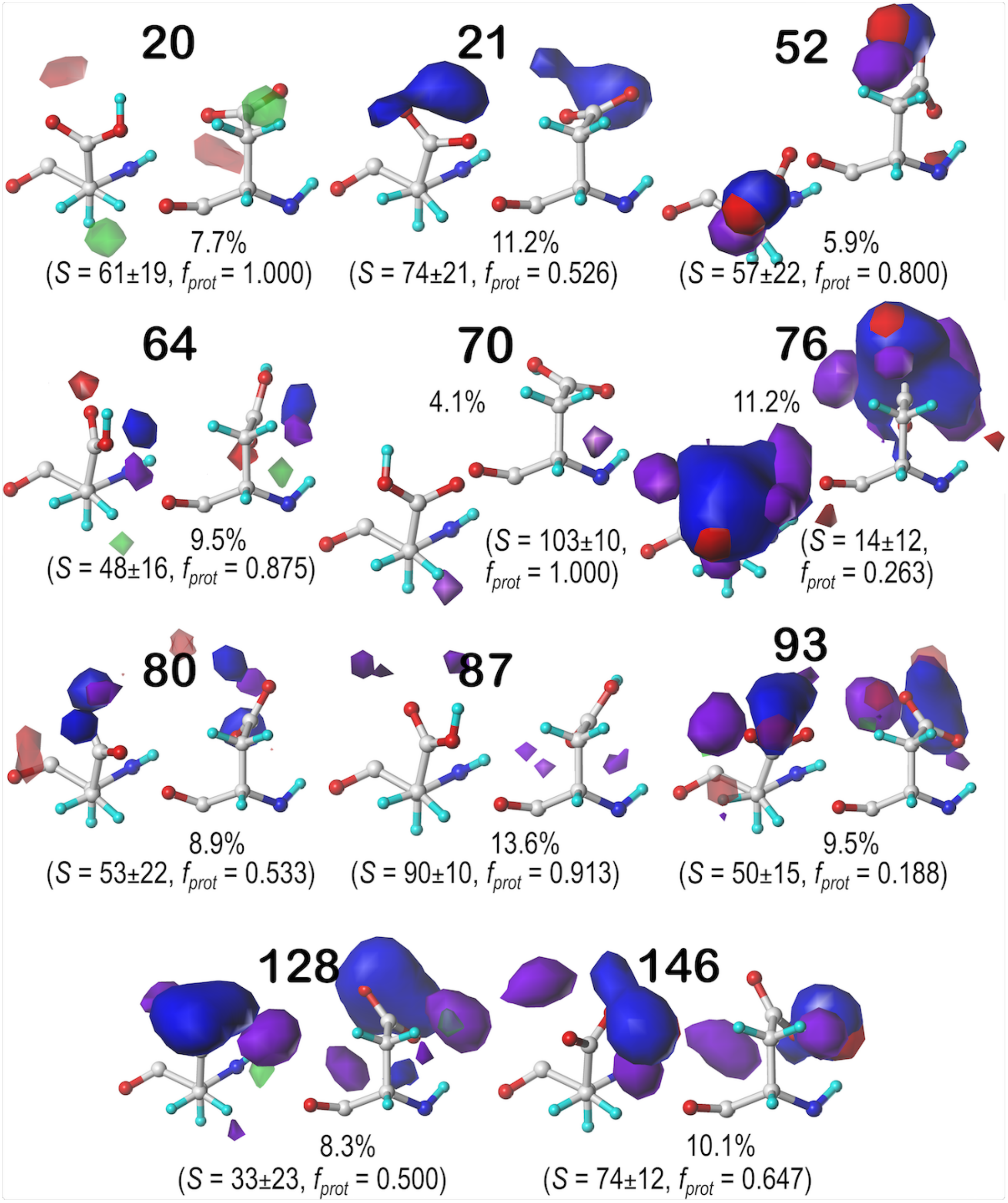

Figure S4: Hydropathic interaction maps displaying the Gaussian-weighted average sidechain environments of aspartic acid in the χ_1 = 60° parse of the *d5* chess square at pH = 3.345. See caption for Figure S1.

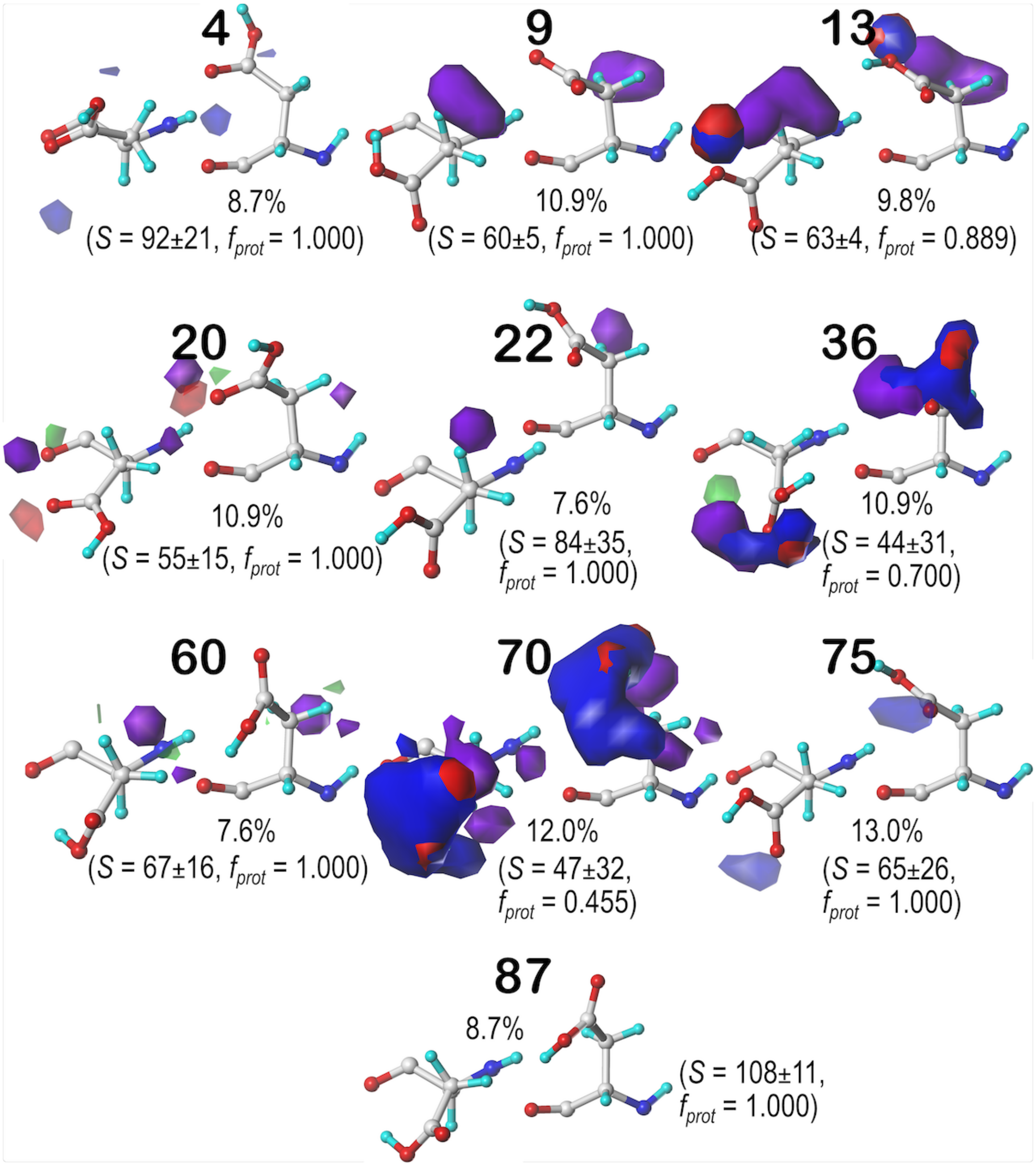

Figure S5: Hydropathic interaction maps displaying the Gaussian-weighted average sidechain environments of aspartic acid in the χ_1 = 180° parse of the *d5* chess square at pH = 3.345. See caption for Figure S1.

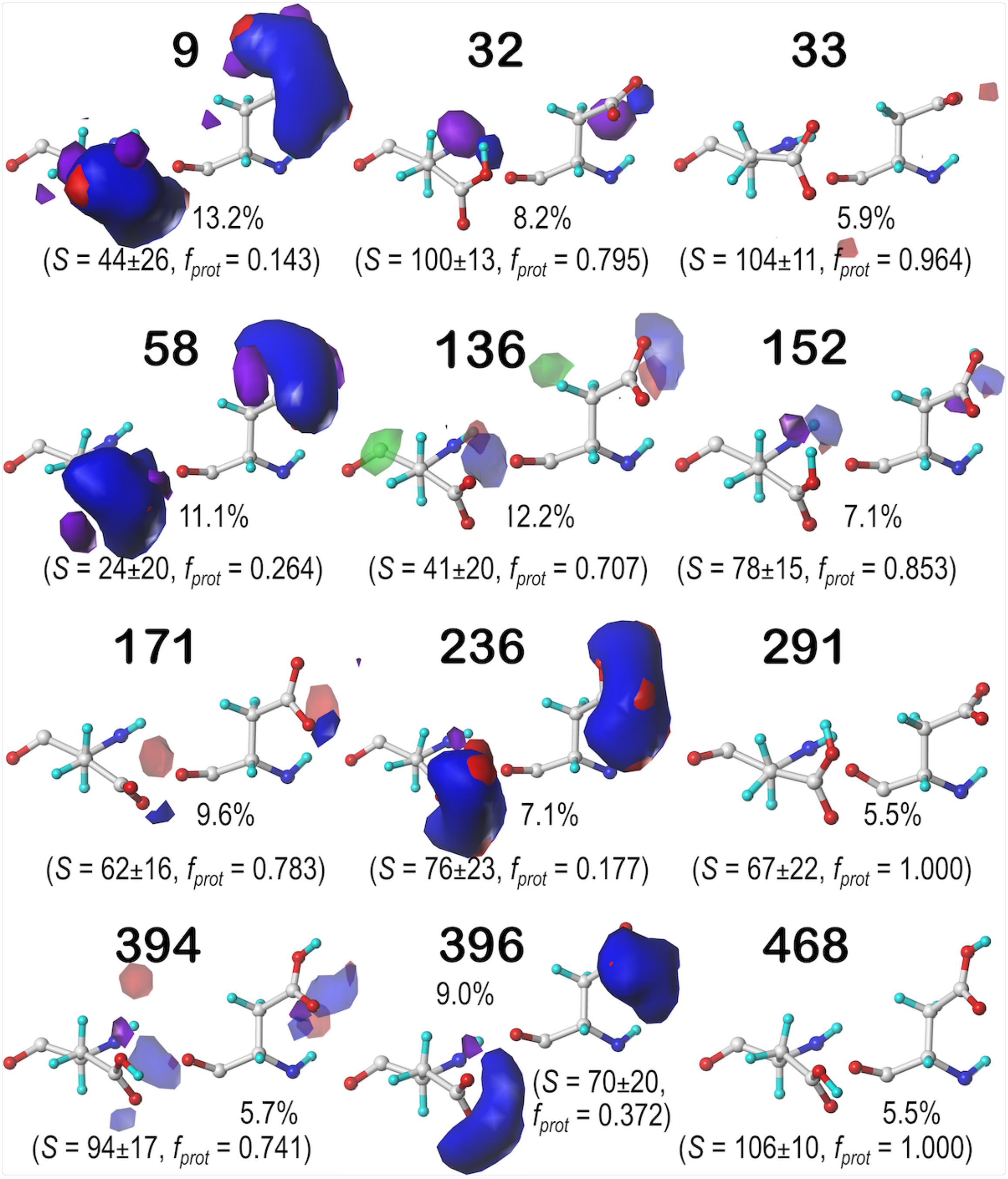

Figure S6: Hydropathic interaction maps displaying the Gaussian-weighted average sidechain environments of aspartic acid in the χ_1 = 300° parse of the *d5* chess square at pH = 3.345. See caption for Figure S1.

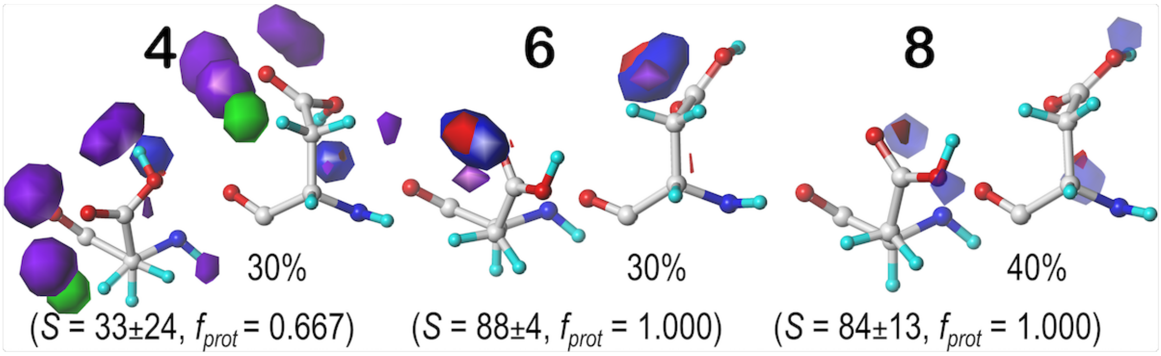

Figure S7: Hydropathic interaction maps displaying the Gaussian-weighted average sidechain environments of aspartic acid in the χ_1 = 60° parse of the *f6* chess square at pH = 3.345. See caption for Figure S1.

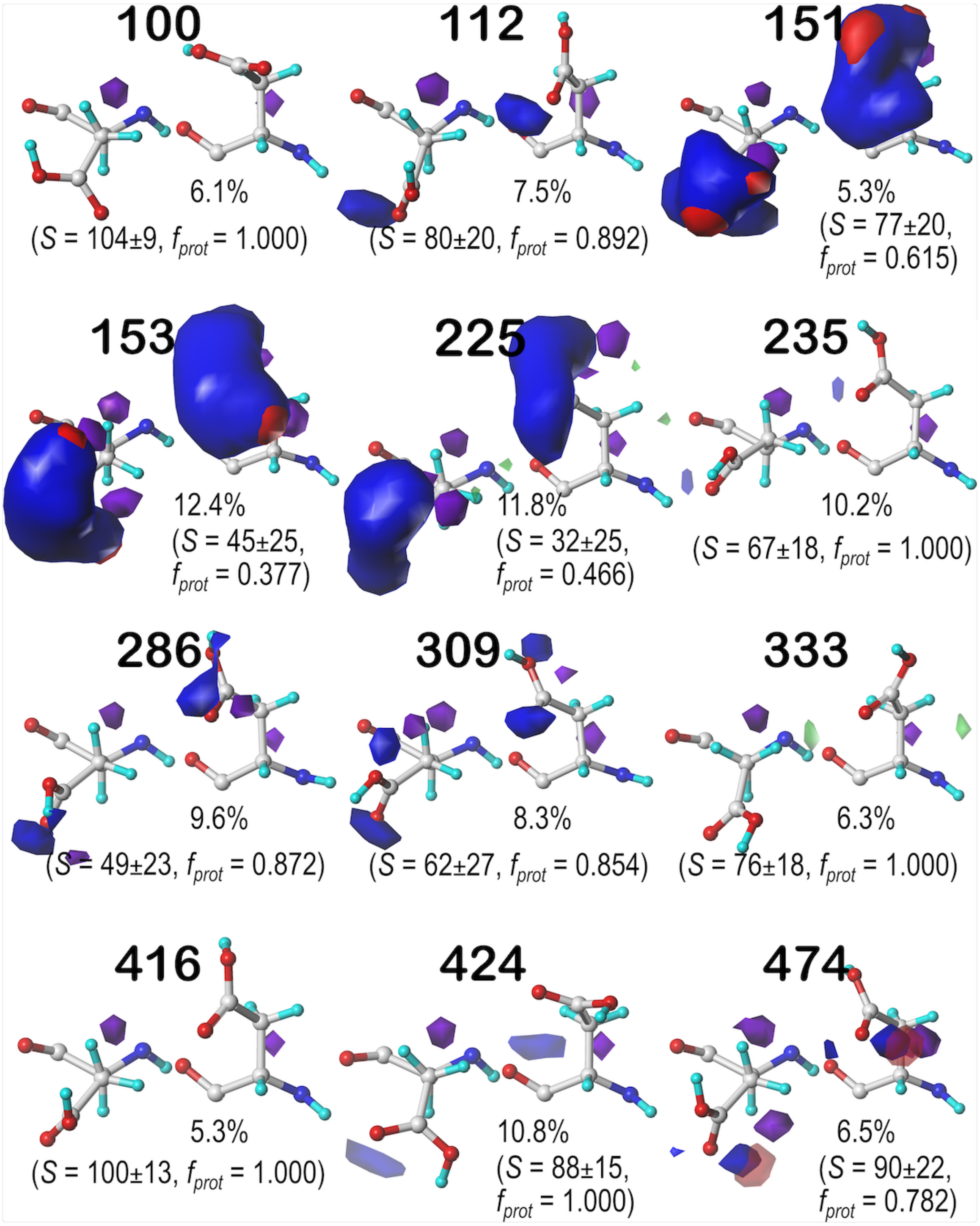

Figure S8: Hydropathic interaction maps displaying the Gaussian-weighted average sidechain environments of aspartic acid in the χ_1 = 180° parse of the *f6* chess square at pH = 3.345. See caption for Figure S1.

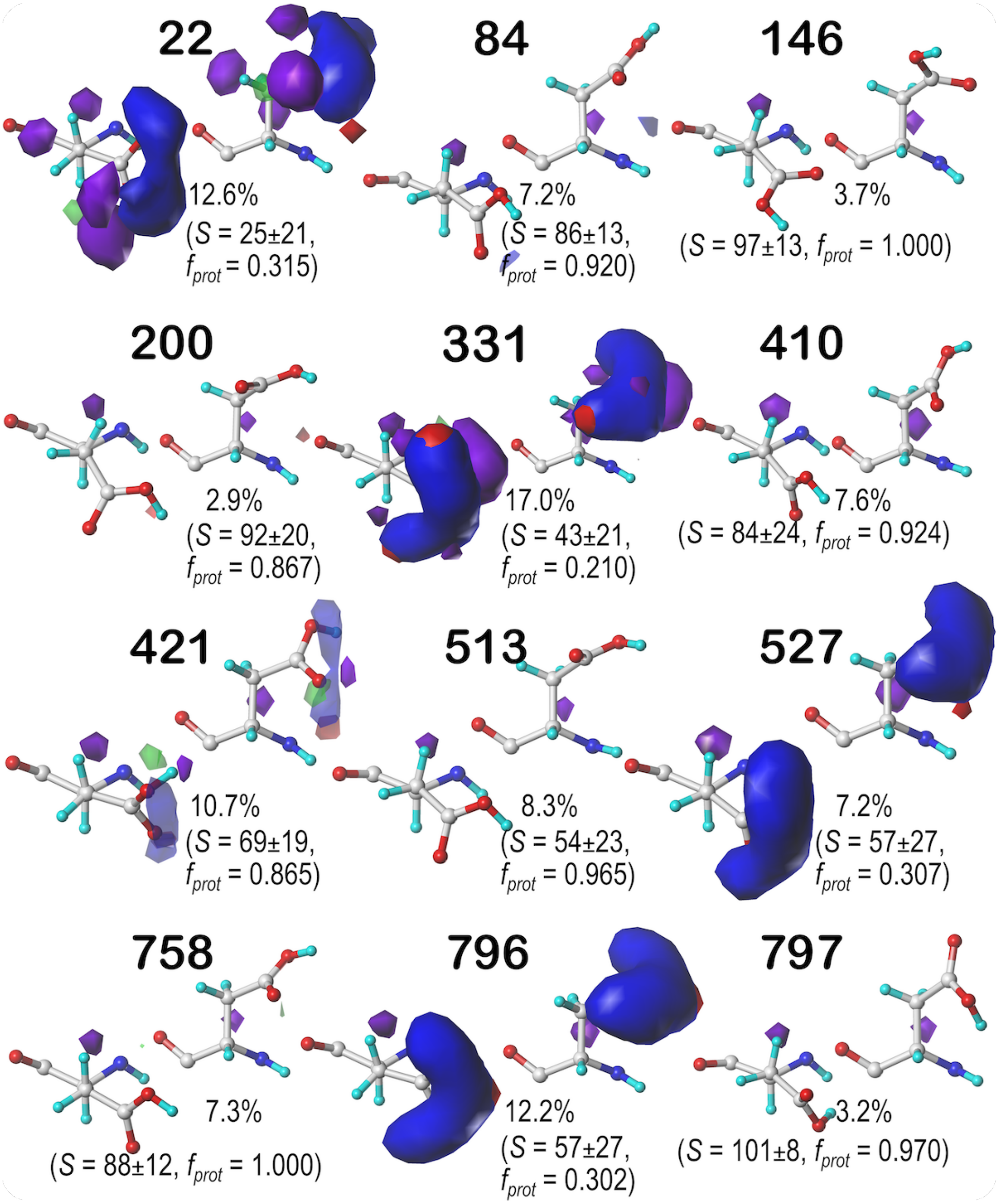

Figure S9: Hydropathic interaction maps displaying the Gaussian-weighted average sidechain environments of aspartic acid in the χ_1 = 300° parse of the *f6* chess square at pH = 3.345. See caption for Figure S1.

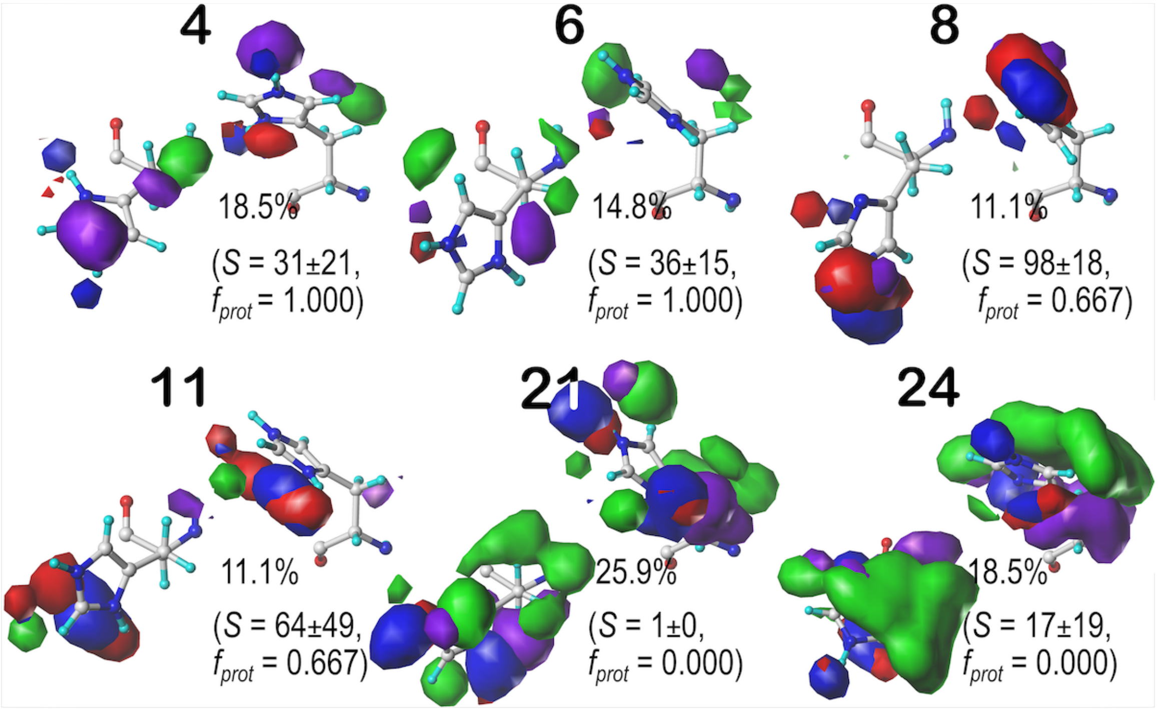

Figure S10: Hydropathic interaction maps displaying the Gaussian-weighted average sidechain environments of histidine in the χ_1 = 180° parse of the *b1* chess square at pH = 5.174. See caption for Figure S1.

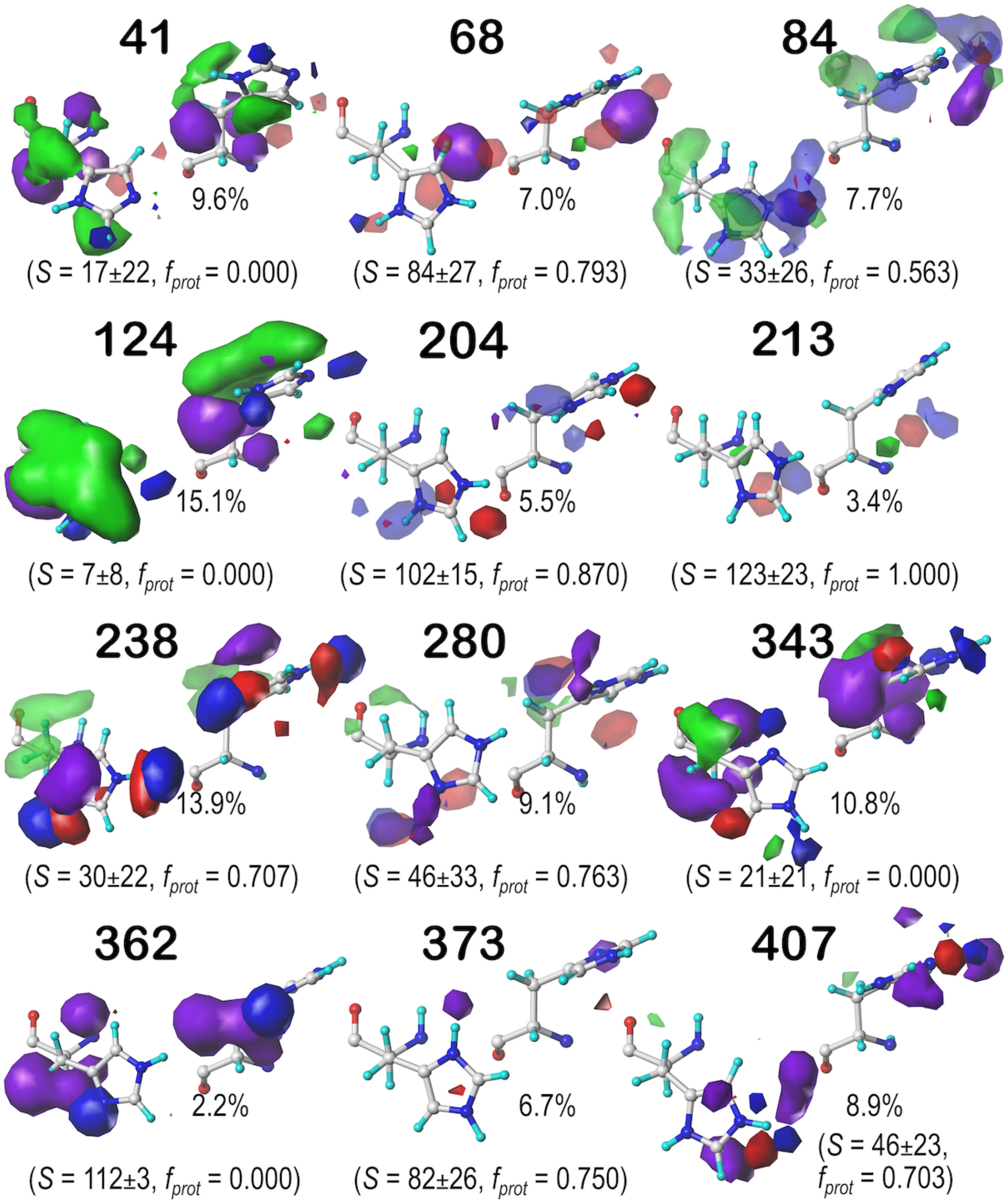

Figure S11: Hydropathic interaction maps displaying the Gaussian-weighted average sidechain environments of histidine in the χ_1 = 300° parse of the *b1* chess square at pH = 5.174. See caption for Figure S1.

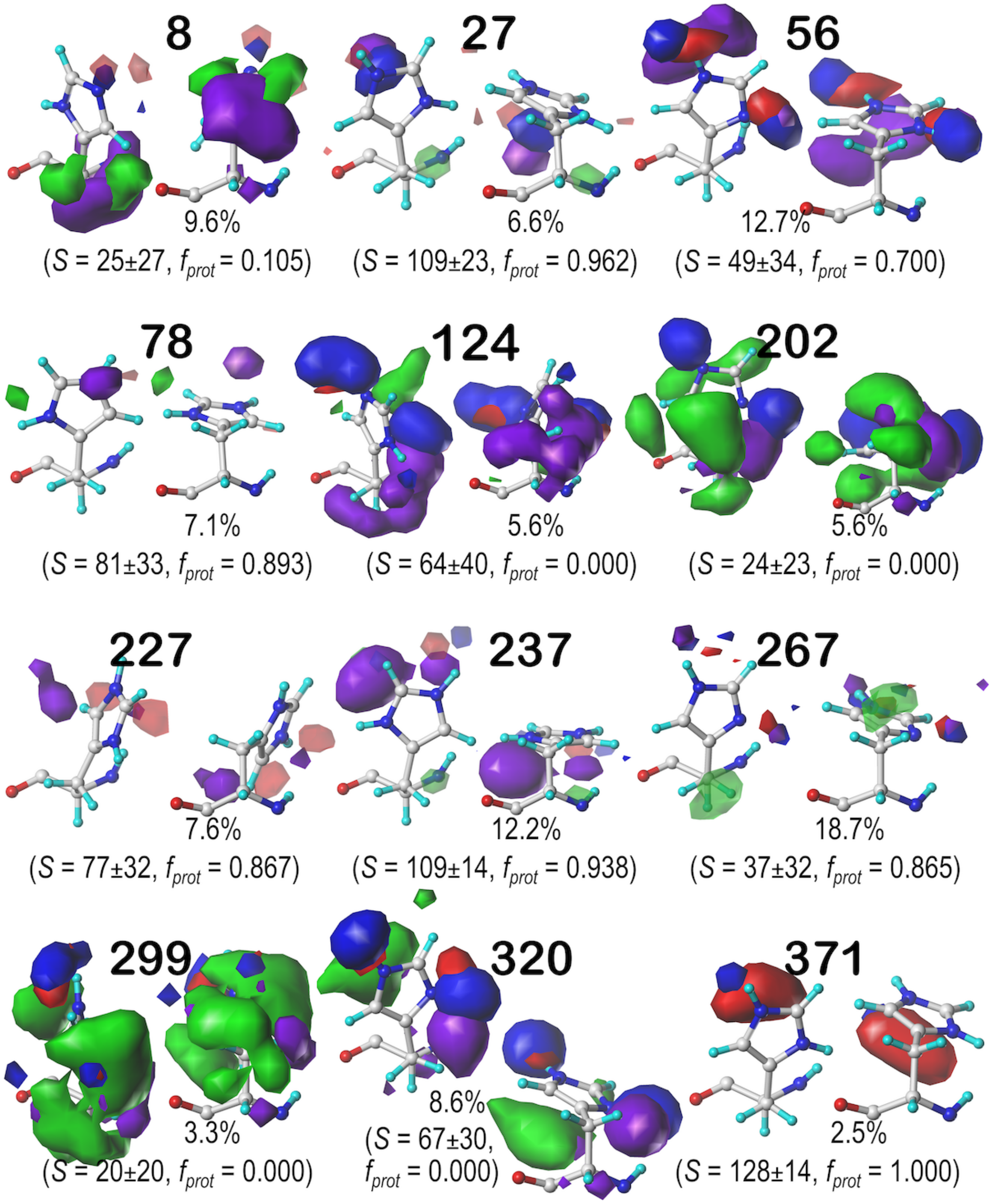

Figure S12: Hydropathic interaction maps displaying the Gaussian-weighted average sidechain environments of histidine in the χ_1 = 60° parse of the *c5* chess square at pH = 5.174. See caption for Figure S1.

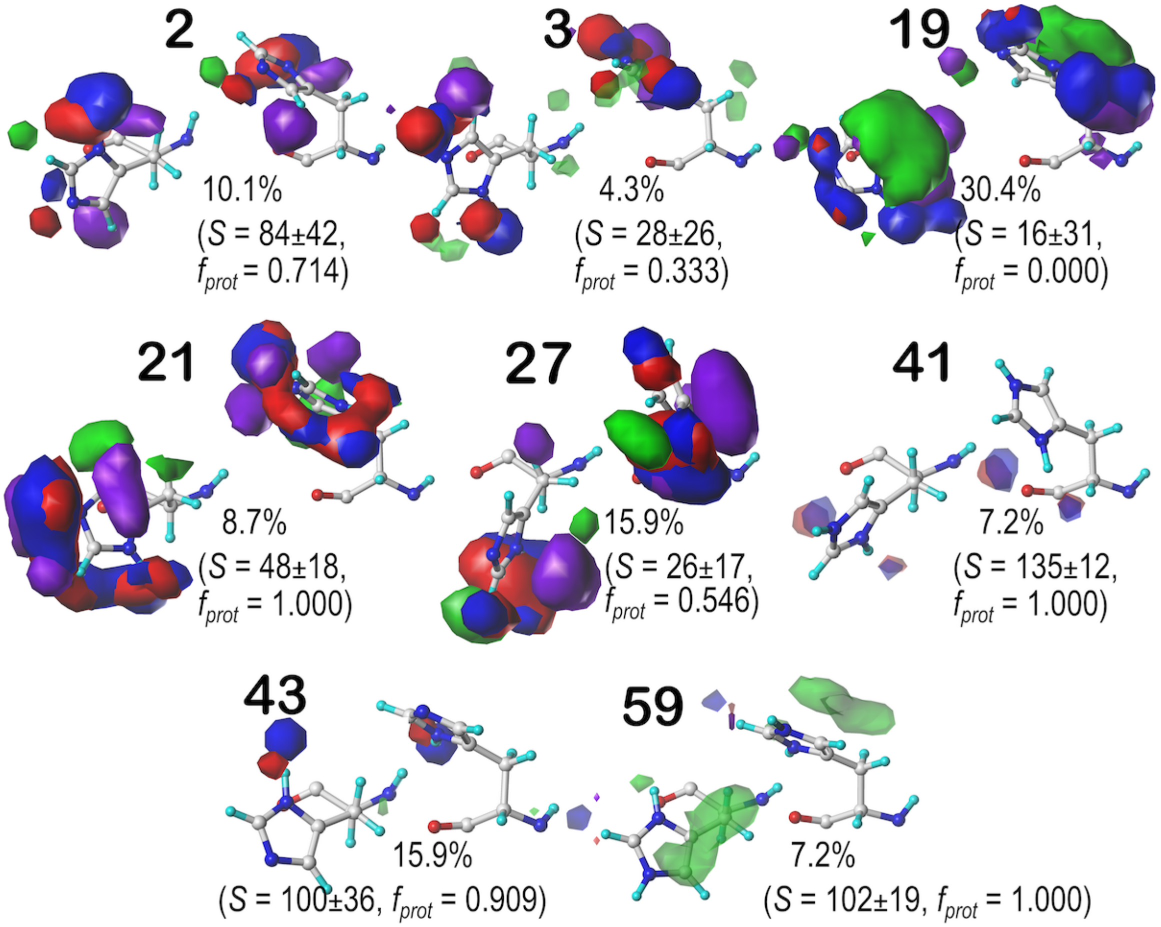

Figure S13: Hydropathic interaction maps displaying the Gaussian-weighted average sidechain environments of histidine in the χ_1 = 180° parse of the *c5* chess square at pH = 5.174. See caption for Figure S1.

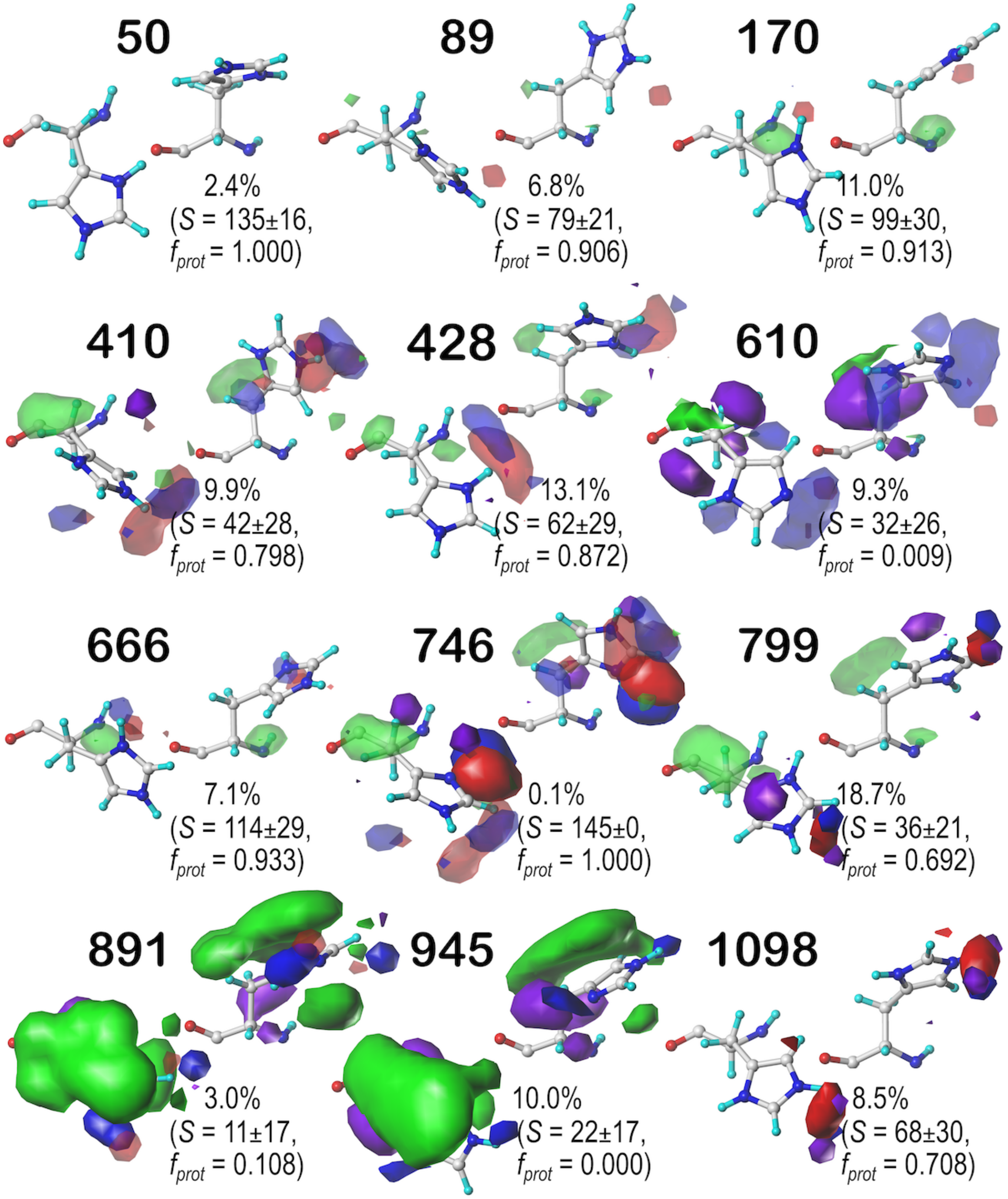

Figure S14: Hydropathic interaction maps displaying the Gaussian-weighted average sidechain environments of histidine in the χ_1 = 300° parse of the *c5* chess square at pH = 5.174. See caption for Figure S1.

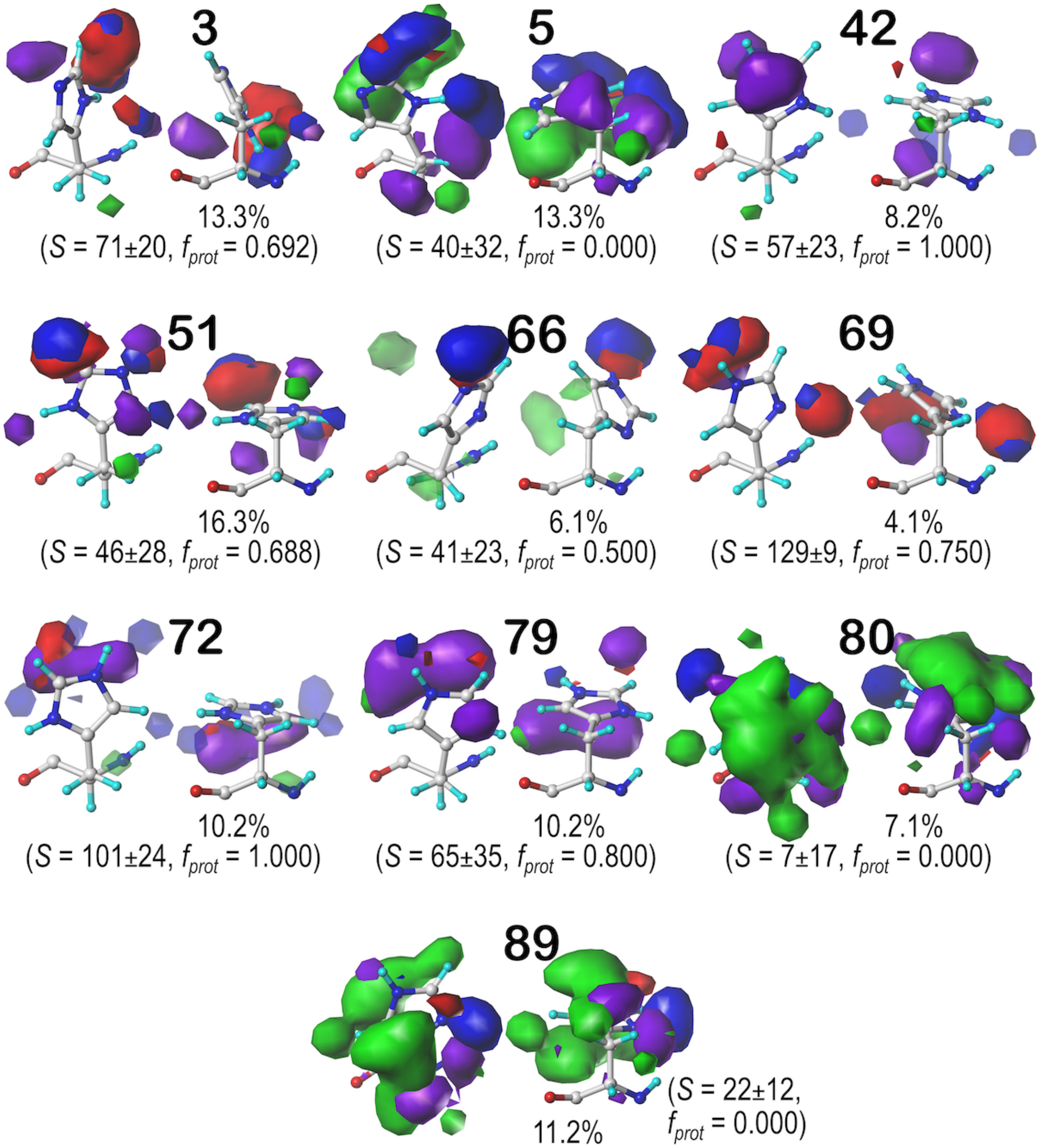

Figure S15: Hydropathic interaction maps displaying the Gaussian-weighted average sidechain environments of histidine in the χ_1 = 60° parse of the *d5* chess square at pH = 5.174. See caption for Figure S1.

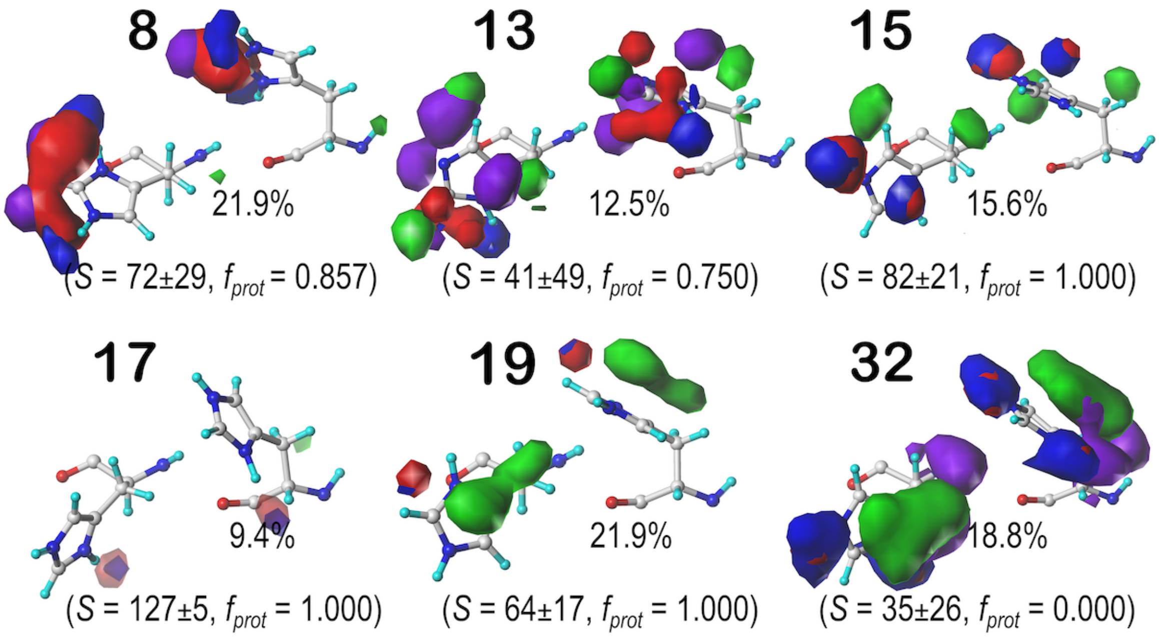

Figure S16: Hydropathic interaction maps displaying the Gaussian-weighted average sidechain environments of histidine in the χ_1 = 180° parse of the *d5* chess square at pH = 5.174. See caption for Figure S1.

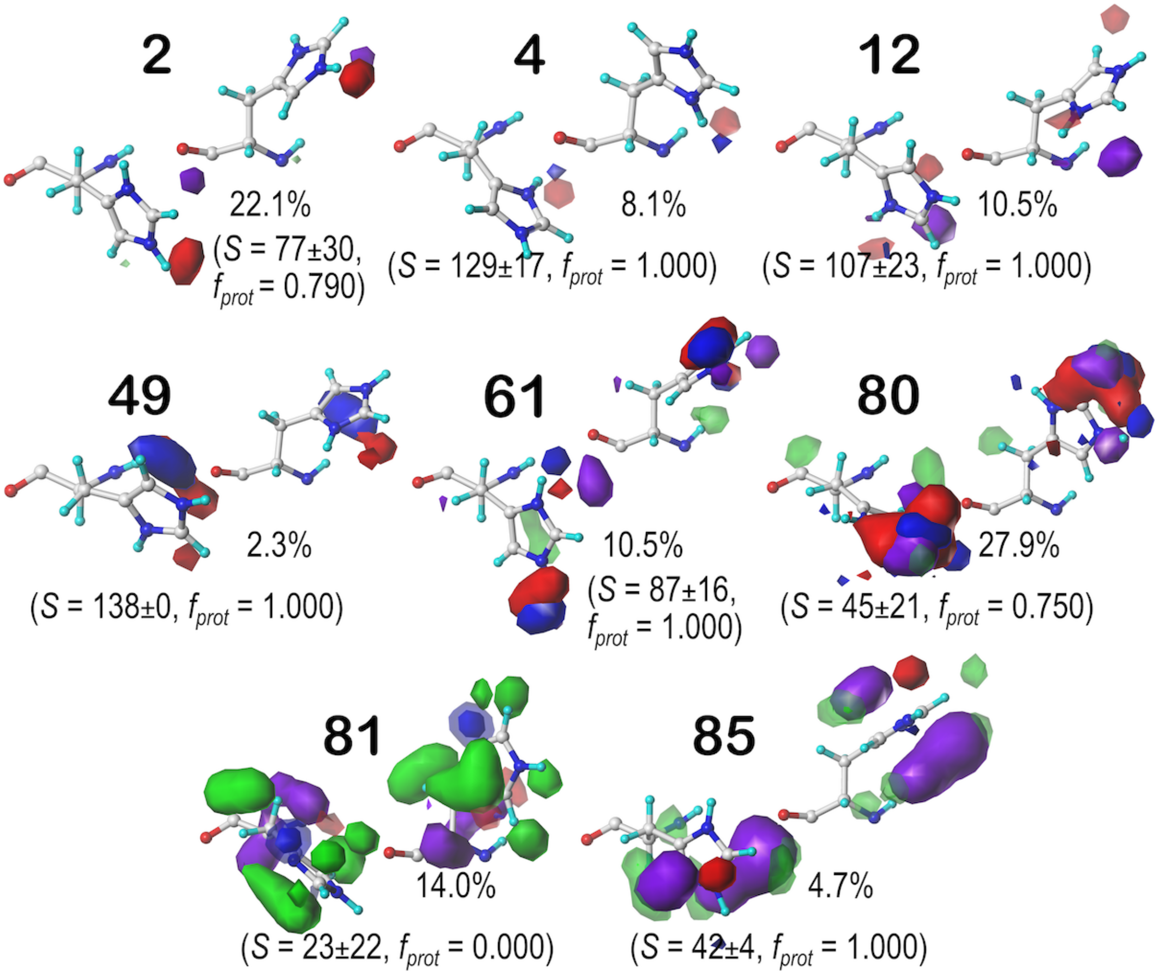

Figure S17: Hydropathic interaction maps displaying the Gaussian-weighted average sidechain environments of histidine in the χ_1 = 300° parse of the *d5* chess square at pH = 5.174. See caption for Figure S1.

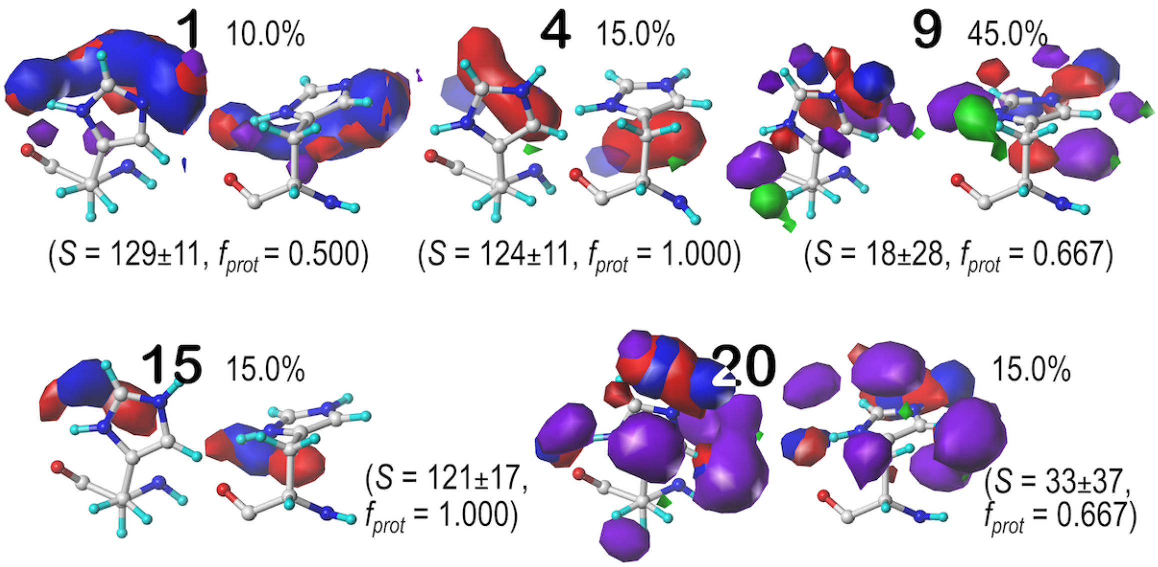

Figure S18: Hydropathic interaction maps displaying the Gaussian-weighted average sidechain environments of histidine in the χ_1 = 60° parse of the *f6* chess square at pH = 5.174. See caption for Figure S1.

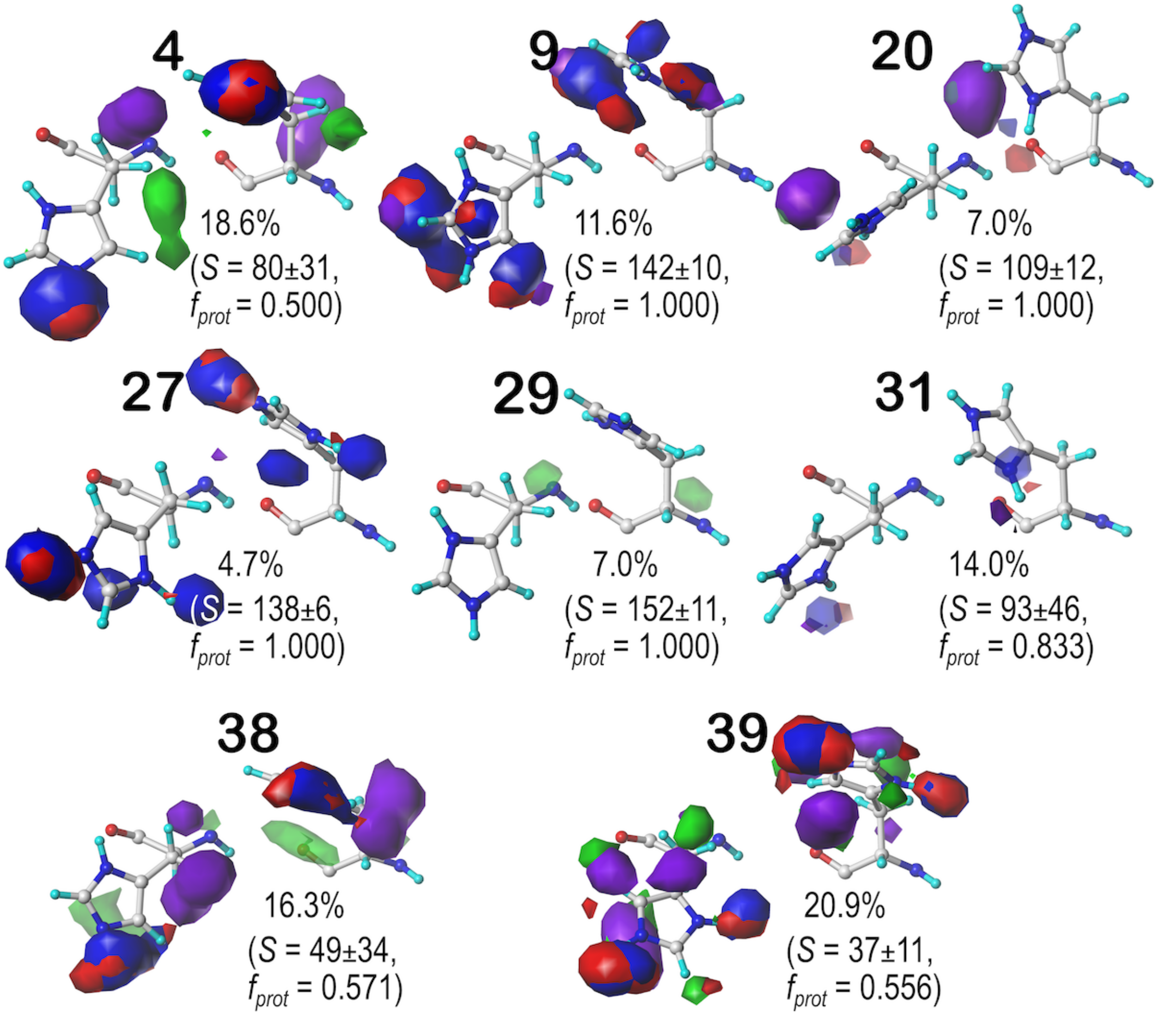

Figure S19: Hydropathic interaction maps displaying the Gaussian-weighted average sidechain environments of histidine in the χ_1 = 180° parse of the *f6* chess square at pH = 5.174. See caption for Figure S1.

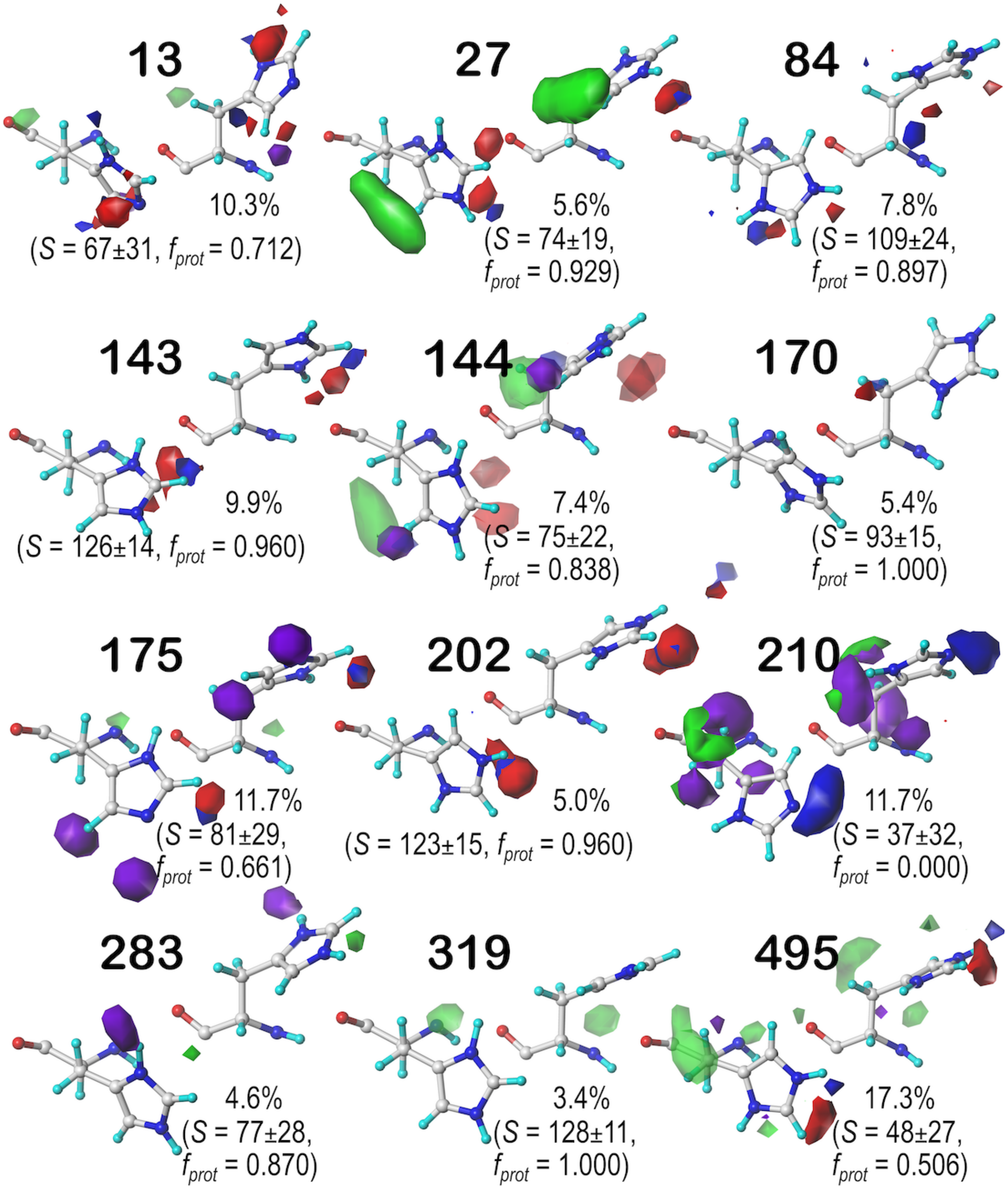

Figure S20: Hydropathic interaction maps displaying the Gaussian-weighted average sidechain environments of histidine in the χ_1 = 300° parse of the *f6* chess square at pH = 5.174. See caption for Figure S1.

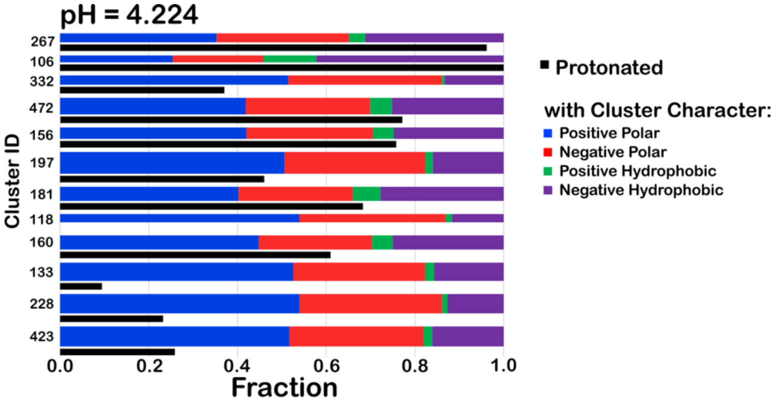

Figure S21: Character interaction chart for the GLU *b1.300.180* parse at pH 4.224. The fraction of each interaction type is given on the x-axis, for each cluster ID on the y-axis. The bars are arranged such that, descending, clusters have smaller SASAs. The thickness of the bars indicates residue population contained within that cluster. The black bars indicate f_{prot}, the fraction of the residues in the cluster protonated.
